# Synergistic Combination of Luteolin and Asiatic Acid on Cervical Cancer In Vitro and In Vivo

**DOI:** 10.3390/cancers15020548

**Published:** 2023-01-16

**Authors:** Ya-Hui Chen, Jyun-Xue Wu, Shun-Fa Yang, Yi-Hsuan Hsiao

**Affiliations:** 1 Women’s Health Research Laboratory, Changhua Christian Hospital, Changhua 50006, Taiwan; 2 Institute of Medicine, Chung Shan Medical University, Taichung 40201, Taiwan; 3 Department of Medical Research, Chung Shan Medical University Hospital, Taichung 40201, Taiwan; 4 Department of Obstetrics and Gynecology, Changhua Christian Hospital, Changhua 50006, Taiwan; 5 School of Medicine, Chung Shan Medical University, Taichung 40201, Taiwan; 6 College of Medicine, Kaohsiung Medical University, Kaohsiung 807378, Taiwan; 7 College of Medicine, National Chung Hsing University, Taichung 40227, Taiwan

**Keywords:** cervical cancer, luteolin, asiatic acid, PI3K/AKT/p70S6K, JNK/p38/ERK, integrin β1/paxillin/FAK

## Abstract

**Simple Summary:**

This study was the first to demonstrate the anticancer effects of luteolin (Lut) combined with asiatic acid (AsA) on CaSki and HeLa cervical cancer cells. Lut combined with AsA effectively reduced cervical cancer cell viability by arresting the cell cycle in the sub-G1 phase and inducing caspase-mediated intrinsic apoptosis. Lut combined with AsA treatment downregulated PI3K/AKT signaling (PI3K, AKT and p70S6K), JNK/p38 MAPK signaling and FAK signaling (integrin β1, paxillin and FAK) and upregulated ERK signaling to induce apoptosis and inhibit cancer cell migration. In in vivo study, Lut combined with AsA markedly inhibited cervical cancer cell-derived xenograft tumor growth and the cytotoxic effect was minimal or nonexistent. Collectively, the present study demonstrated that Lut combined with AsA may be used as an anticancer agent in future clinical treatment to improve the prognosis of cervical cancer.

**Abstract:**

Cervical cancer is an important issue globally because it is the second most common gynecological malignant tumor and conventional treatment effects have been shown to be limited. Lut and AsA are plant-derived natural flavonoid and triterpenoid products that have exhibited anticancer activities and can modulate various signaling pathways. Thus, the aim of the present study was to evaluate whether Lut combined with AsA could enhance the anticancer effect to inhibit cervical cancer cell proliferation and examine the underlying molecular mechanisms in vitro and in vivo. The results of a CCK-8 assay showed that Lut combined with AsA more effectively inhibited the proliferation of CaSki and HeLa cells than Lut or AsA treatment alone. Lut combined with AsA caused apoptosis induction and sub-G1-phase arrest in CaSki and HeLa cells, as confirmed by flow cytometry, mitoROS analysis, antioxidant activity measurement and western blot assay. In addition, Lut combined with AsA significantly inhibited the cell migration ability of CaSki and HeLa cells in a wound-healing assay. Furthermore, Lut combined with AsA induced apoptosis and inhibited migration through downregulated PI3K/AKT (PI3K, AKT and p70S6K), JNK/p38 MAPK and FAK (integrin β1, paxillin and FAK) signaling and upregulated ERK signaling. In an in vivo study, Lut combined with AsA markedly inhibited cervical cancer cell-derived xenograft tumor growth. Collectively, the present study showed that Lut combined with AsA may be used as an anticancer agent to improve the prognosis of cervical cancer. Indeed, with additional research to develop standardized dosages, Lut and AsA combination therapy could also be applied in clinical medicine.

## 1. Introduction

Cervical cancer is one of the major gynecologic malignancies, with more than 600,000 women diagnosed and 342,000 deaths in 2020 [1]. Conventional treatment has a limited efficacy and is prone to cause drug resistance, recurrence and metastasis, and thus the outcome is not favorable; the overall 5-year survival rate is only 66.7% for advanced cervical cancer patients [2]. Several studies have shown that approximately 90% of cervical carcinomas are associated with high-risk human papillomavirus (HPV) infection, especially HPV16 and HPV18, which cause 70–72% of invasive cervical cancers [3,4,5]. Thus, new treatment agents to inhibit HPV-linked carcinogenesis, improve the chemotherapy outcome and enhance the overall survival rate of cervical cancer patients need to be developed.

In recent years, scientists and researchers have discovered that plant-derived natural products with antitumor activities can inhibit tumor cell proliferation, induce apoptosis and reverse multidrug resistance in cancer therapy and lower the toxic effect on normal cells [6,7,8]. Thus, we investigated the therapeutic effects and mechanisms of luteolin (Lut) and asiatic acid (AsA) on cervical cancer. AsA (2,3, 23-trihydroxy-urs-12-ene-28-oic acid, C_30_H_48_O_5_) is a natural triterpenoid compound and the major source extracted from Centella asiatica [9] and has been shown to exert cardioprotective [10], neuroprotective [11,12], anti-diabetes [13], anti-inflammation [14], antioxidant [15], antimicrobial [16] and wounding-healing effects [17]. Dong et al. reported that AsA could inhibit BMMs’ cell osteoclastogenesis and alleviate ovariectomy-induced bone loss by downregulating the signaling pathway of NF-κB/MAPK/Akt [18]. Previous studies have demonstrated the anti-tumor effects of AsA in terms of inhibiting cell growth and inducing apoptosis in several tumors, such as ovarian [19], lung [20], colon [21], nasopharyngeal carcinoma [22], breast [23,24], osteosarcoma [25] and renal cancer [26]. Moreover, several molecular pathways, such as Pl3K/AKT/mTOR, VEGF/VEGFR2, AMPK-dependent, JAK2/STAT3 and ERK/p38/JNK signaling, have been found to mediate the biological activity of AsA [19,20,21,22,23,24,25,26]. AsA combined with naringenin synergistically inhibits melanoma and lung carcinoma progression by promoting natural killer (NK) cell development and inactivating TGF-β1/Smad3 signaling [27]. Li et al. [28], Jing et al. [29] and Gonçalves et al. [30] showed that an anticancer effect was observed upon exclusive use of AsA in cervical cell line HeLa cells via cell cycle arrest and the mitochondrial apoptosis pathway; however, the biological effect and exact anticancer molecular mechanism of AsA in cervical cancer remain unclear.

Lut (3’,4’,5,7-tetrahydroxyflavone, C_15_H_10_O_6_) is a natural flavonoid present in different plants species, such as vegetables, fruits and medicinal herbs [31,32]. Numerous studies have demonstrated that Lut has various biological effects and protective functions, including anti-inflammatory, antioxidant, anti-allergic, immune-modulation, anti-microbiota and anti-atherosclerosis effects [33,34,35,36]. Additionally, Lut has been observed to possess the ability to obstruct the development of various cancers in vitro and in vivo by inhibiting cancer cell proliferation, activating cell cycle arrest, protecting carcinogenic stimuli, inducing apoptosis and suppressing cancer metastasis through different signaling pathways [37,38,39,40]. Wu et al. [41] and Tsai et al. [42] revealed that Lut can effectively inactivate the AKT/mTOR pathway and reverse epithelial–mesenchymal transition (EMT) to suppress breast cancer cell proliferation and metastasis, as well as downregulating Nrf2-mediated expression to enhance chemosensitivity in breast cancer treatment. Potočnjak et al. [43] also indicated that the antitumor activity of Lut in colon cancer SW620 cells acted through the ERK/FOXO3a-dependent mechanism and had anti-metastatic potential. Previous studies also revealed that Lut could inhibit the proliferation of HeLa cervical cancer cells through G2/M arrest, apoptosis and upregulation of the p16^INK4A^ and JNK expression and downregulation of TNF-α-induced NF-κB activation [44,45]. Lut showed a synergistic effect with TNF-related apoptosis-inducing ligand (TRAIL), which could induce apoptosis in HeLa cells by activation of death receptor 5 and caspase-8 [46]. These results suggested that Lut may be a potentially effective anticancer agent for the treatment of cervical cancer. Based on these results, this study assessed whether Lut can enhance the anticancer effect of AsA on cervical cancer cells and further evaluated the underlying mechanism using both cervical cancer cells (CaSki, HeLa and C33A) and a xenograft mouse model.

## 2. Materials and Methods

### 2.1. Chemicals

Asiatic acid (AsA, #HY-N0194, ≥99.47% purity) and luteolin (Lut, #HY-N0162, ≥98.42% purity) were purchased from MedChemExpress (Monmouth, NJ, USA), suspended in dimethyl sulfoxide (DMSO, D26650; Sigma-Aldrich, St. Louis, MO, USA) to prepare the stock solution (50 mM) and kept at −20 °C for further use. Z-Val-Ala-Asp-fluoromethylketone (z-VAD-fmk), an inhibitor of pan-caspase, was purchased from BioVision, Inc. (Milpitas, CA, USA).

### 2.2. Cell Culture

Human cervical cancer cell lines CaSki, HeLa and C33A were purchased from the Bioresource Collection and Research Center (BCRC, Hsinchu, Taiwan; derived from ATCC^®^ CRM-1550^TM^, ATCC^®^ CRM-CCL-2^TM^ and ATCC^®^ CRM-HTB-31^TM^). Cells were cultured in RPMI 1640 medium (1-41P05-K; BioConcept, Amimed, Allschwil, Switzerland) or Eagle’s minimum essential medium (EMEM, SH30024.02; Cytiva, Marlborough, MA, USA) containing 10% fetal bovine serum (FBS, SH30396.03; Cytiva), 0.1 Mm non-essential amino acids (NEAA, SH30238.03; Cytiva), 1 mM sodium pyruvate (SH30239.01; Cytiva) and 1% penicillin/streptomycin/amphotericin B (SV30079.01; Cytiva) in a humidified incubator with 5% CO_2_ at 37 °C.

### 2.3. Cell Viability and Drug Combination Assay

CaSki, HeLa and C33A cells were seeded into 96-well plates with 2 × 10^4^ cells per well and then treated with different concentrations of Lut (10, 50, 75 and 100 μM) with or without AsA (25, 50 and 75 μM) for 24 h. To each well, 90 μL fresh culture medium and 10 μL CCK-8 solution (cell counting kit-8, #CK04; Dojindo Molecular Technique, Inc., Rockville, MD, USA) were added and cells were incubated at 37 °C for 1 h. Absorbance was measured at 450 nm using a FLUOstar Galaxy microplate reader (BMG Labtech, Ortenberg, Germany). The effect of combination was evaluated using the combination index (CI) method of Chou and Talalay and CalcuSyn software (Biosoft, Cambridge, UK) [47]. Generally, CI < 1 indicates synergy, CI = 1 indicates additivity and CI > 1 indicates antagonism. Cells treated with 0.1% DMSO in culture medium were used as controls and were regarded as 100% viable and the viabilities of the Lut-treated or AsA-treated cells were determined. The half-inhibitory concentration (IC50) values were calculated as the drug concentration that inhibited cell proliferation by 50% compared to vehicle controls. In further experiments, cells were pretreated with z-VAD-fmk (#1009-20C, 2.5 μmol/L, BioVision, Inc.) prior to Lut and AsA treatment.

### 2.4. Apoptosis Analysis

CaSki and HeLa cells were seeded into 6-well plates with 1 × 10^6^ cells per well and treated with 50 μM Lut with or without 50 or 75 μM AsA for 24 h. Then, an FITC Annexin V apoptosis detection kit (#556547, BD Biosciences, Bergen, NJ, USA) was used to detect 10,000 collected cells in different stages of cell death; cells were harvested and suspended in 1× Annexin V binding buffer (100 uL) and then double-stained with 5 μL FITC Annexin V (20 μg/mL) and 5 μL propidium iodide (PI, 50 μg/mL) in the dark for 15 min at room temperature (RT), adding 1x Annexin V binding buffer (900 uL) to each tube. Finally, stained cells were analyzed by Cytomics FC500 flow cytometry and CXP software (version 2.3; Beckman Coulter, Inc., Brea, CA, USA). Early and late apoptotic/necrotic cells were assessed and quantified.

### 2.5. Cell Cycle Analysis

CaSki and HeLa cells were seeded into 6-well plates with 1 × 10^6^ cells per well and treated with 50 μM Lut with or without 50 or 75 μM AsA for 24 h. Cells were then collected and fixed with 70% ice-cold ethanol (1 mL) at −20 °C overnight (16–18 h). After fixation, the cells were centrifuged at 400× *g* at 4 °C for 10 min, washed with cold PBS and stained with 0.5 mL BD Pharmingen^TM^ PI/RNases staining buffer (PI, 10 μg/mL; RNases, 300 μg/mL; #550825; BD Biosciences) in the dark for 15 min at RT (25 °C). Finally, cell cycle distribution data were collected for 10,000 collected cells by Cytomics FC500 flow cytometry and CXP software (version 2.3; Beckman Coulter, Inc.).

### 2.6. Mitochondrial ROS Measurement

CaSki and HeLa cells were seeded into 6-well plates with 1 × 10^6^ cells per well and treated with 50 μM Lut with or without 50 or 75 μM AsA for 24 h. The cells were harvested and suspended in RPMI 1640 medium or EMEM medium (0.5 mL) and then the cells were immediately supplemented with 2.5 uL of 0.5 mM MitoSOX Red mitochondrial superoxide indicator solution (#M36008; Invitrogen, Thermo Fisher Scientific Inc., Waltham, MA, USA; the final concentration of MitoSOX = 2.5 μM) and incubated in a 37 °C water bath for 20 min in the dark. The cells washed with 37 °C prewarmed PBS, submitted to centrifugation to pellet the cells and then resuspended in warm PBS buffer (0.5 mL). Fluorescence signal data were obtained using a Cytomics FC 500 flow cytometer (Beckman Coulter, Inc.) and analyzed using FlowJo software (version 7.6; BD Life Sciences, Franklin Lakes, NJ, USA).

### 2.7. Measurement of Glutathione (GSH) and Catalase Levels

The glutathione (GSH) and catalase levels of the cell lysate were determined using an Amplite^TM^ Fluorimetric Glutathione assay kit (#10055; AAT Bioquest^®^, Inc., Sunnyvale, CA, USA) and an Amplite^TM^ Fluorimetric Catalase assay kit (#11306; AAT Bioquest^®^, Inc.), respectively. CaSki and HeLa cells were seeded into 6-well plates with 1 × 10^6^ cells per well and treated with 50 μM Lut with or without 50 or 75 μM AsA for 24 h. PBS was used to wash and suspend cells, followed by centrifugation for 10 min at 400× *g*. Collected cells were resuspended in 0.1% Triton X-100 (250 μL), followed by centrifugation for 7 min at 380× *g* and further centrifugation at 16,000× *g* for 1 min. Supernatant (50 μL) was added onto the 96-well microliter plate, followed by 50 μL Thiolite^TM^ Green reaction mixture and the plate was incubated at RT for 30 min in the dark. Absorbance was measured at Ex/Em = 490/520 nm using a CLARIOstar fluorescence microplate reader (BMG Labtech, Ortenberg, Germany). To measure the total catalase activity, 50 μL supernatant was applied onto a 96-well microliter plate, and 50 μL H_2_O_2_ (10 μM) assay buffer was added, followed by incubation at RT for 30 min in the dark, after which 50 μL catalase assay mixture (1× Amplite^TM^ Red and 100 mU/mL horseradish peroxidase) was applied. After 30 min, the absorbance was measured at Ex/Em = 540/590 nm using a CLARIOstar fluorescence microplate reader (BMG Labtech).

### 2.8. Wound-Healing Assay

CaSki and HeLa cells were cultured in 6-well plates with 1 × 10^6^ cells per well to form a confluent monolayer and straight wounds were made by scratching with a 200-μL pipette tip. After washing with medium to remove cell debris, the wounded monolayers were treated with 50 μM Lut with or without 50 or 75 μM AsA for 0, 12 and 24 h. The wound gaps were photographed at regular intervals (0, 12 and 24 h) under an Olympus BX61 microscope (Olympus Corporation, Shinjuku, Tokyo, Japan) at 100× magnification, and the cell-free wound areas were measured using ImageJ software (http://rsb.info.nih.gov/ij, accessed on 11 May 2022, NIH, Bethesda, MD, USA). To reduce variability in the results, multiple views of each well were documented, and wound closure was evaluated as a percentage relative to the untreated control.

### 2.9. Western Blot Analysis

CaSki and HeLa cells were seeded into 10-cm dishes with 2 × 10^6^ cells per dish, pre-treated with or without z-VAD-fmk (2.5 μM) at 37 °C for 2 h, and then treated with 50 μM Lut with or without 50 μM AsA for 24 h. Next, proteins were extracted from the cells using RIPA buffer (#20-188; Millipore, Billerica, MA, USA) and protein concentrations were quantified using a BCA protein assay kit (#23225; Thermo Fisher Scientific Inc.). Whole-cell lysates (30 µg) were separated on 10–12% (*w*/*v*) SDS-PAGE and transferred to 0.2-µm PVDF membranes (EA162-0177; Bio-Rad, Irvine, CA, USA). Following blocking with BlockPRO^TM^ protein blocking buffer (#BF01-1L; Energenesis Biomedical Co., Ltd.) for 1 h, incubation with the indicated primary antibodies (1:1000 dilution) was performed at 4 °C overnight: PARP (#9532), Bcl-2 (#15071), Bax (#5023), cleaved caspase-3 (#9664), phospho-AKT (Ser473) (#4060), Akt (#4298), phospho-p70S6 (T389) (#9234), p70S6 (#2708), phospho-P38 (#9211), P38 (#9212), phospho-ERK1/2 (#4370), ERK1/2 (#4695), phospho-JNK (#4668) and JNK (#9258) were purchased from Cell Signaling (Danvers, MA, USA); PIK3CA (NBP2-19804; Novus, CO, USA); phospho-FAK (GTX129840), FAK (GTX100764), integrin β1 (GTX128839) and paxillin (GTX129840) were purchased from GeneTex (Irvine, CA, USA); and GAPDH (MA5-15738; Thermo Fisher Scientific Inc.). Horseradish peroxidase (HRP)-conjugated goat anti-mouse (#115-035-003, 1:50,000 dilution) or anti-rabbit (#111-035-003; 1:100,000 dilution; Jackson ImmunoResearch, Laboratories, Inc. West Grove, PA, USA) IgG polyclonal secondary antibody was added to the membranes for 1h at RT. Protein bands were detected using an enhanced chemiluminescence reagent (#WBKLS0500; ECL, Millipore, Burlington, MA, USA) and relative intensity calculations were performed using Fusion-Capt Advanced FX7 software (version 16.08a; Labtech International, Inc., Vilber Lourmat, France).

### 2.10. Xenograft Mouse Model

Seven-week-old female BALB/cAnN.Cg-*Foxn1^nu^*/CrlNarl (NUDE) mice were purchased from the National Laboratory Animal Center (Taipei, Taiwan). CaSki cells (1 × 10^7^) were mixed with Corning^®^ Matrigel^®^ Matrix reagent (#354248; Corning Inc., Tewksbury, MA, USA) at a 2:1 ratio and injected into the right flank of the mice. Tumor volume (mm^3^) and body weight were measured using an electronic caliper every two days, using the following standard formula: Volume = [(length) × (width)^2^]/2. When the tumor volume reached ~130 mm^3^ (Day 6), the mice were randomly divided into four groups (six mice per group) and drug treatment was initiated via daily intraperitoneal injection (i.p.): vehicle control group [10% DMSO, 40% Cremophor/ethanol (3:1; Sigma-Aldrich) and 50% PBS]; AsA group [100 mg/kg/day, dissolved in 10% DMSO, 40% Cremophor/ethanol (3:1) and 50% PBS]; Lut group (50 mg/kg/day, dissolved in 10% DMSO, 40% Cremophor/ethanol and 50% PBS); and combination group (asiatic acid 100 mg/kg/day plus luteolin 50 mg/kg/day). The mouse tumors were extracted for tissue section preparation and the tumor weight was recorded after sacrifice on day 22. Cancer cell implantation was performed using a 2–3% isoflurane (Panion & BF Biotech) inhalation, and the mice sacrifice was performed using a CO_2_ chamber. The animal protocols were approved by the Institutional Animal Care and Use Committee (IACUC) of Changhua Christian Hospital, Taiwan (Approval No: CCH-AE-109-020).

### 2.11. Histopathology and Immunohistochemistry Analyses

Fresh tumor tissue was fixed with 10% neutral buffered formalin (#3800600; Leica Biosystems Richmond, Inc. Richmond, IL, USA) and embedded in paraffin. Sections (5-μm-thick) were stained with hematoxylin and eosin (#3801698; Leica Biosystems Richmond, Inc.) and subjected to immunohistochemical (IHC) analysis. Sections were de-waved, rehydrated, boiled in ddH_2_O for 10 min, then 3% H_2_O_2_ for 10 min, 3% BSA (A3294, Sigma-Aldrich, in PBS) for 1 h and stained for ki67 (#12202; 1:400) and cleaved caspase-3 (#9664; 1:100), purchased from Cell Signaling; and integrin β1 (GTX128839; 1:100), phospho-FAK (GTX129840; 1:200) and paxillin (GTX129840; 1:100) purchased from GeneTex were used at 4 °C overnight. Sections were washed with PBS and incubated with OneStep Polymer HRP-conjugated anti-mouse/rat/rabbit IgG secondary antibodies (GTX83398; GeneTex) at RT for 30 min. Sections were then visualized using a colorimetric reagent DAB detection kit (GTX30939; GeneTex). Nuclei were counterstained with hematoxylin (#3801522; Leica Biosystems Richmond, Inc.) and photographed using an Olympus BX61 microscope (Olympus Corporation). For each animal tissue specimen, four fields at 400× magnification were evaluated using Image-Pro Plus 4.5 software (Media Cybermetics, Silver Spring, MD, USA).

### 2.12. Statistical Analysis

Statistical analyses were performed using the one-way ANOVA followed by Bonferroni correction’s post-test using GraphPad software (version 9.3.0; Dotmatics, Inc., San Diego, CA, USA). All data representing results are presented as the mean ± standard deviation (SD) from at least three independent experiments. A *p* value < 0.05 was considered to indicate a statistically significant difference.

## 3. Results

### 3.1. Luteolin and Asiatic Acid Inhibited the Proliferation of Cervical Cancer Cell Lines

**Figure 1:**
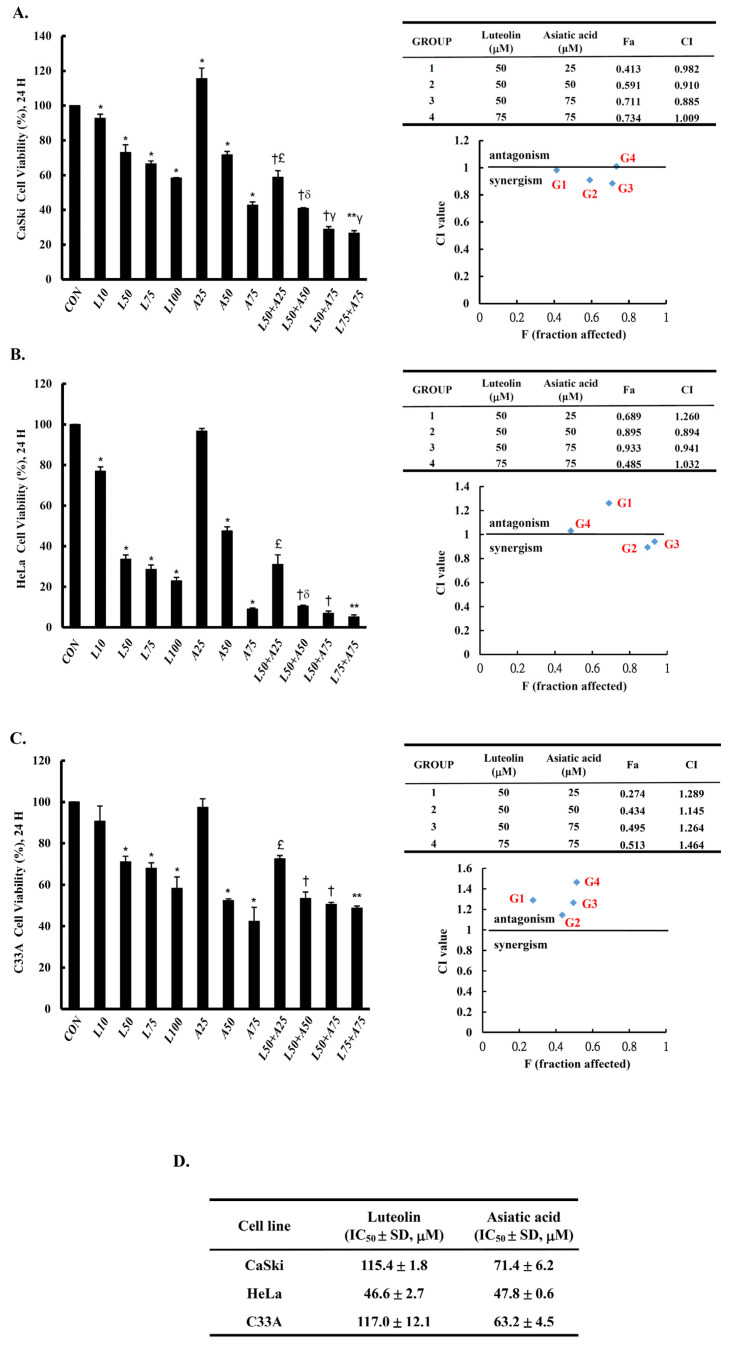
Luteolin and asiatic acid inhibited the proliferation of cervical cancer cell lines. CaSki (**A**), HeLa (**B**) and C33A (**C**) cell proliferation was measured using a CCK-8 assay after cells were exposed to Lut or AsA alone or in combination. (**D**) IC50 values for luteolin and asiatic acid treatments in cervical cancer cells. Values represent mean ± SD from three replicates. *, †, **, £, δ and γ *p* ˂ 0.05 compared with CON, L50-, L75-, A25-, A50- or A75-treated group. CON, 0.1% DMSO; L10, 10 μM luteolin; L50, 50 μM luteolin; L75, 75 μM luteolin; L100, 100 μM luteolin; A25, 25 μM asiatic acid; A50, 50 μM asiatic acid; A75, 75 μM asiatic acid. CI, combination index, IC_50_, half maximal inhibitory concentration. G1, 50 μM luteolin + 25 μM asiatic acid; G2, 50 μM luteolin + 50 μM asiatic acid; G3, 50 μM luteolin + 75 μM asiatic acid; G4, 75 μM luteolin + 75 μM asiatic acid; blue mark, combination index values.

To assess the anti-proliferation effects of both Lut and AsA on cervical cancer cells, a CCK-8 assay was performed using CaSki, HeLa and C33A cells with various concentrations of Lut (0–100 μM) with or without AsA (0–75 μM) for 24 h. We observed that Lut and AsA dose-dependently decreased the proliferation of CaSki, HeLa and C33A cells (Figure 1, *p* < 0.05; and Figure S1 for 48 and 72 h), although 25 μM AsA did not, in the case of CaSki cells. Moreover, we also found that Lut had a lower IC_50_ value, of 46.6 ± 2.7 μM for HeLa cells, than CaSki and C33A cells, which had values of 115.4 ± 1.8 μM and 117.0 ± 12.1 μM, respectively. The IC_50_ values for the AsA treatment were found to be 47.8 ± 0.6 μM for HeLa cells, 71.4 ± 6.2 μM for CaSki cells and 63.2 ± 4.5 μM for C33A cells. Lut was less active than AsA, which showed more strong antiproliferative activity against CaSki cells and C33A cells. In addition, we further investigated cell proliferation under a combination of Lut and AsA. Compared with the control cells (100%), the proliferation of CaSki cells was significantly decreased by 28% and 57% after treatment with 50 and 75 μM AsA (71.6 ± 2.1% vs. 42.8 ± 1.8%) and inhibited by 59% and 71% when treated in combination with 50 μM Lut (40.9 ± 0.3% vs. 28.9 ± 1.5%), respectively. The 75 μM AsA combined with 75 μM Lut (26.7 ± 1.4%) also had approximately 73% inhibition efficiency. Similarly, HeLa cell proliferation significantly decreased by 52% and 91% after treatment with 50 and 75 μM AsA (47.5 ± 2.1% vs. 9.0± 0.6%) and was markedly inhibited, by 89% and 93%, when combined with 50 μM Lut (47.5 ± 2.1% vs. 9.0± 0.6%), respectively. The 75 μM AsA combined with 75 μM Lut (5.1 ± 1.0%) had 95% inhibition efficiency. Additionally, C33A cell proliferation significantly decreased, by 48% and 58%, after treatment with 50 and 75 μM AsA (52.4 ± 0.8% vs. 42.3± 6.9%) and was inhibited by 47% and 49% when combined with 50 μM Lut (53.4 ± 3.1% vs. 50.5 ± 1.0%), respectively. The 75 μM AsA combined with 75 μM Lut (48.8 ± 0.9%) only had 51% inhibition efficiency. Additionally, it can be seen from comparison of CI values, AsA combined with Lut exerted synergistic effects, with the CI being 0.910 and 0.885 for 50 or 75 μM AsA combined with 50 μM Lut for CaSki cells; and 0.894 and 0.941 for 50 or 75 μM AsA combined with 50 μM Lut for HeLa cells; and 1.145 and 1.264 for 50 or 75 μM AsA combined with 50 μM Lut for C33A cells, respectively. Therefore, AsA combined with Lut was more effective in reducing cell proliferation of CaSki and HeLa cells relative to the control cells and to cells treated with Lut or AsA alone, but C33A cells did not show a synergistic effect.

### 3.2. Luteolin and Asiatic Acid Induced Cell Apoptosis in Cervical Cancer Cell Lines

**Figure 2:**
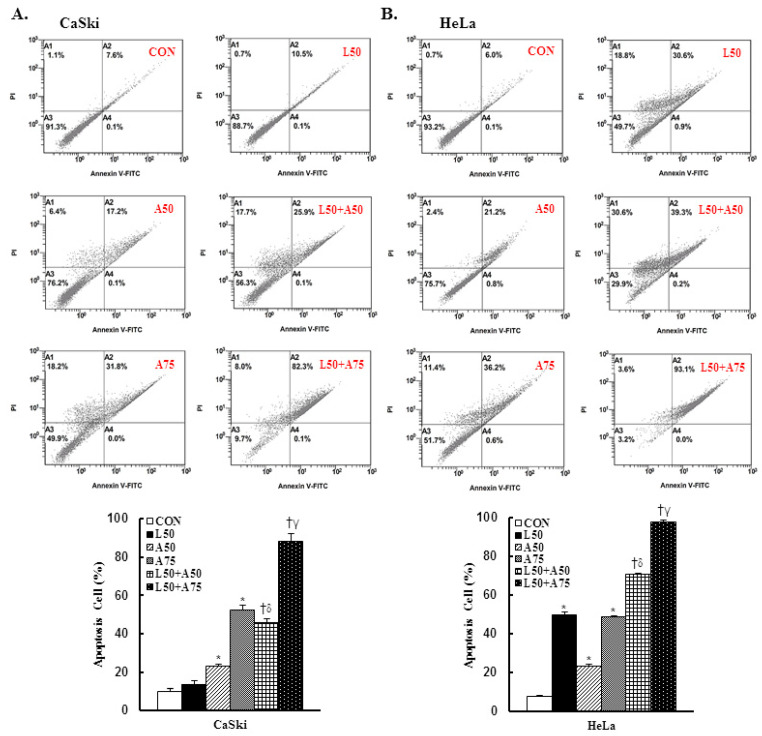
The combination of luteolin and asiatic acid induced greater apoptosis in CaSki and HeLa cells. CaSki and HeLa cells were analyzed using flow cytometry after 24 h of treatment with Lut (50 μM) or AsA (50 and 75 μM) alone or in combination. Representative flow cytometry dot plots (upper panel) and histograms (lower panel) reveal cell apoptosis (Annexin V- and PI-stained cells) and quantitative analysis for (**A**) CaSki and (**B**) HeLa cells. Values represent mean ± SD from three replicates. *, †, δ and γ *p* ˂ 0.05 compared with CON, L50-, A50- or A75-treated group. CON, 0.1% DMSO; L50, 50 μM luteolin; A50, 50 μM asiatic acid; A75, 75 μM asiatic acid. L50 + A50, 50 μM luteolin + 50 μM asiatic acid; L50 + A75, 50 μM luteolin + 75 μM asiatic acid.

To determine whether apoptosis induction was involved in the anti-proliferation activity of Lut and AsA on cervical cancer cells, CaSki and HeLa cells were subjected to Annexin V-FITC and PI staining by flow cytometry. As shown in Figure 2, as compared with the control group, Lut treatment increased the number of apoptotic cells (CaSki cells, 13.4 ± 2.1% vs. 9.9 ± 1.5%; HeLa cells, 49.9 ± 1.1% vs. 7.4 ± 0.6%) and AsA resulted in a significant increase in a dose-dependent manner (CaSki cells, 23.3 ± 0.8% vs. 52.4 ± 2.5%; HeLa cells, 23.0 ± 1.0% vs. 48.5 ± 0.9%). As expected, Lut (50 μM) combined with AsA (50 and 75 μM) more significantly increased the apoptotic cell percentages (CaSki cells, 45.6 ± 2.0% vs. 88.1 ± 4.1%; HeLa cells, 70.6 ± 0.6% vs. 97.8 ± 1.0%, respectively) as compared with Lut or AsA alone (*p* < 0.05). In summary, combined Lut and AsA promoted greater apoptosis of CaSki and HeLa cells.

### 3.3. Luteolin and Asiatic Acid Induced Cell Cycle Arrest in Cervical Cancer Cell Lines

**Figure 3:**
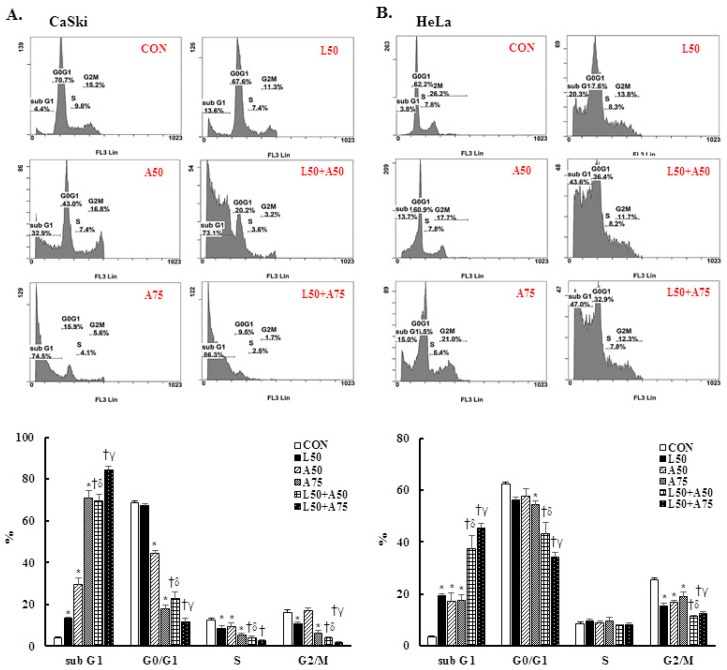
The combination of luteolin and asiatic acid led to cell cycle arrest in the sub-G1 phase in CaSki and HeLa cells. CaSki and HeLa cells were analyzed using flow cytometry after 24 h of treatment with Lut (50 μM) or AsA (50 and 75 μM) alone or in combination. Representative flow cytometry results (upper panel) and histograms (lower panel) reveal the cell cycle distribution (sub-G1, G0/G1, S and G2/M) and quantitative analysis for (**A**) CaSki and (**B**) HeLa cells. Values represent mean ± SD from three replicates. *, †, δ and γ *p* ˂ 0.05 compared with CON, L50-, A50- or A75-treated group. CON, 0.1% DMSO; L50, 50 μM luteolin; A50, 50 μM asiatic acid; A75, 75 μM asiatic acid. L50 + A50, 50 μM luteolin + 50 μM asiatic acid; L50 + A75, 50 μM luteolin + 75 μM asiatic acid.

To understand the anti-proliferation effects of Lut and AsA, cell cycle progression analysis of cervical cancer cells was performed by flow cytometry. In CaSki cells, Lut or AsA treatment significantly increased the sub-G1 phase cells, from 3.9 ± 0.5% (untreated cells) to 13.8 ± 0.2% (50 μM Lut), to 29.5 ± 3.1% (50 μM AsA) and to 70.8 ± 3.6% (75 μM AsA) and significantly decreased the G0/G1 phase cells, from 68.4 ± 1.2% (untreated cells) to 67.2 ± 0.8% (50 μM Lut), to 44.4 ± 1.4% (50 μM AsA) and to 18.0 ± 1.9% (75 μM AsA). Simultaneously, the percentage of sub-G1 phase cells increased from 3.6 ± 0.2% (untreated cells) to 19.3 ± 0.9% (50 μM Lut), to 17.3 ± 3.1% (50 μM AsA) and to 17.4 ± 2.3% (75 μM AsA) and decreased the G0/G1 phase cells from 62.6 ± 0.5% (untreated cells) to 56.4 ± 1.1% (50 μM Lut), to 57.8 ± 2.8% (50 μM AsA), and to 54.5 ± 1.3% (75 μM AsA) in HeLa cells (Figure 3, *p* < 0.05). Furthermore, we found that treatment with Lut (50 μM) combined with AsA (50 and 75 μM) resulted in significantly higher percentages of sub-G1 phase cells (CaSki cells, 69.4 ± 3.2% vs. 84.1 ± 1.9%; HeLa cells, 37.5 ± 4.9% vs. 45.6 ± 1.8%) and lower percentages of G0/G1 phase cells (CaSki cells, 23.1 ± 2.7% vs. 11.7 ± 1.9%; HeLa cells, 43.3 ± 4.4% vs. 34.2 ± 1.9%) than in cells treated with Lut or AsA alone (*p* < 0.05). These results suggested that Lut combined with AsA induced more effective cell cycle arrest in the sub-G1 phase.

### 3.4. Luteolin and Asiatic Acid Upregulated Mitochondrial ROS (mitoROS) and Downregulated Glutathione (GSH) in Cervical Cancer Cell Lines

**Figure 4:**
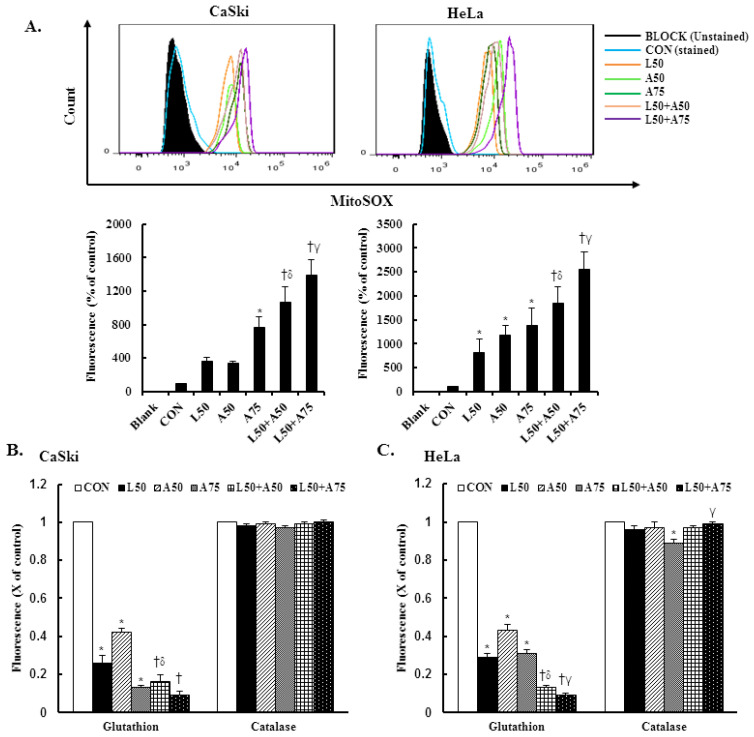
The combination of luteolin and asiatic acid upregulated mitoROS production and downregulated GSH activation. CaSki and HeLa cells were treated with Lut (50 μM) or AsA (50 and 75 μM) alone or in combination for 24 h. (**A**) Flow cytometry was used to analyze the mitochondrial ROS level and quantitative results are presented in the lower plot. (**B**,**C**) GSH and catalase activation were analyzed using ELISA. Quantitative results are shown in the lower plot. Values represent mean ± SD from three replicates. *, †, δ and γ *p* ˂ 0.05 compared with CON, L50-, A50- or A75-treated group. CON, 0.1% DMSO; L50, 50 μM luteolin; A50, 50 μM asiatic acid; A75, 75 μM asiatic acid. L50 + A50, 50 μM luteolin + 50 μM asiatic acid; L50 + A75, 50 μM luteolin + 75 μM asiatic acid.

Excessive mitochondrial ROS has been shown to induce cell injury and death and induce apoptosis or necroptosis. Mitochondrial antioxidants have been reported to be effective in cancer prevention and anticancer therapy. Therefore, we examined mitochondrial ROS and mitochondrial antioxidant expressions using the MitoSOX Red reagent and an enzyme-linked immunosorbent assay (ELISA). The results revealed that the MitoSOX Red-derived fluorescence level was significantly increased at 50 μM Lut,= and 50 or 75 μM AsA in CaSki and HeLa cells (CaSki cells, 363.8 ± 49.4% vs. 334.5 ± 30.0% vs. 764.5 ± 123.6%; HeLa cells, 813.8 ± 286.1% vs. 1183.3 ± 201.6% vs. 1386.2 ± 363.2%, respectively) as compared with the non-treated control cells (100%, Figure 4A, *p* < 0.05). In addition, combined treatment with Lut and AsA further significantly increased the MitoSOX Red-derived fluorescence levels of CaSki and HeLa cells (CaSki cells, 1069.0 ± 187.2% vs. 1387.2 ± 190.6%; HeLa cells, 1848.7 ± 339.2% vs. 2562.2 ± 354.3%, respectively) as compared with cells treated with Lut or AsA alone. In addition, when compared with the non-treated control, Lut or AsA monotherapy significantly reduced the GSH level in CaSki and HeLa cells (CaSki cells, ratio of control: 0.26 ± 0.04 vs. 0.42 ± 0.02 vs. 0.13 ± 0.01; HeLa cells, ratio of control: 0.29 ± 0.02 vs. 0.43 ± 0.03 vs. 0.31 ± 0.01, respectively, Figure 4B,C, *p* < 0.05); Lut in combination with AsA more significantly reduced the GSH level (CaSki cells, ratio of control: 0.16 ± 0.04 vs. 0.09 ± 0.02; HeLa cells, ratio of control: 0.13 ± 0.01 vs. 0.09 ± 0.01, respectively). The catalase expression level did not change in CaSki and HeLa cells. Thus, these data showed that Lut combined with AsA treatment may induce cervical cancer cell apoptosis via upregulating mitoROS and downregulating the GSH level to promote cell death.

### 3.5. Luteolin and Asiatic Acid Inhibited Cell Migration of Cervical Cancer Cells

**Figure 5:**
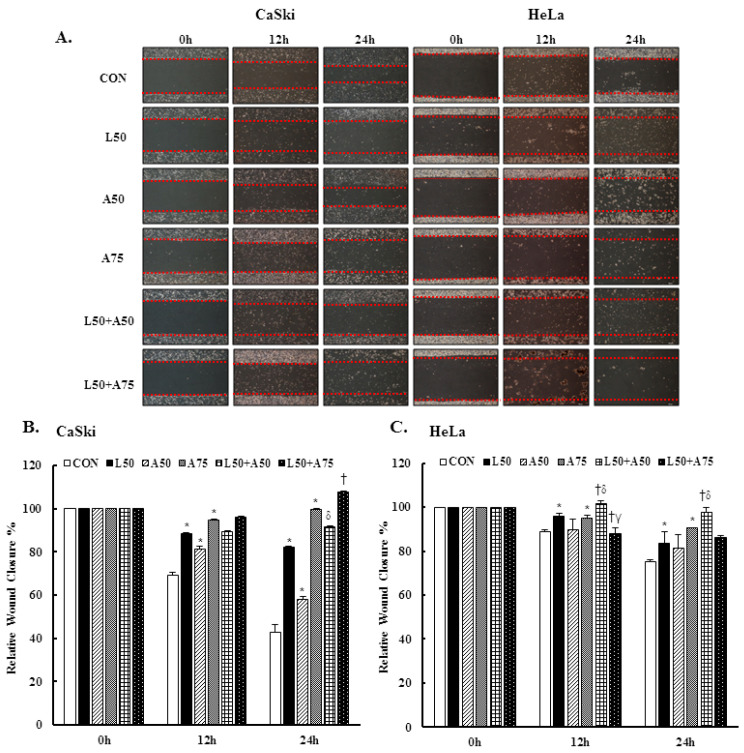

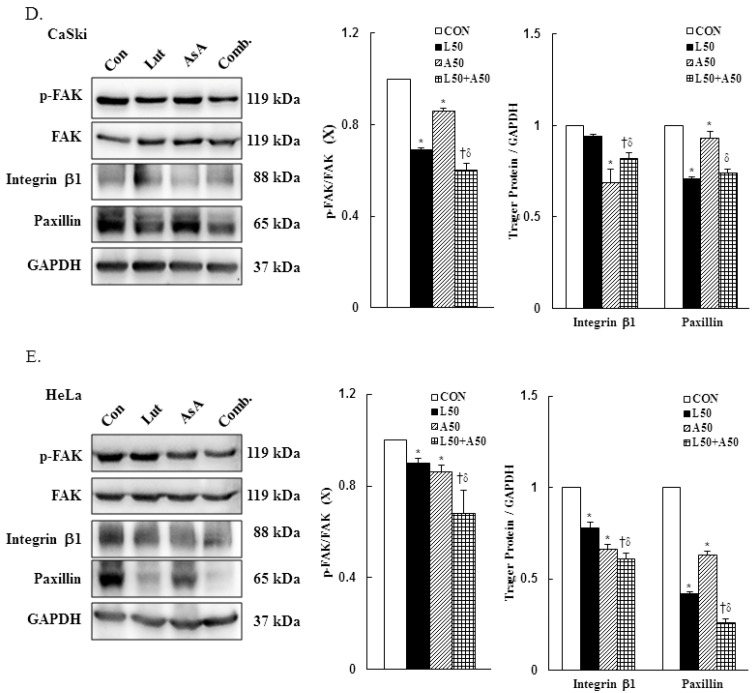
Luteolin combined with asiatic acid inhibited cell migration and inactivated the integrin β1/FAK/paxillin pathway. CaSki and HeLa cells were treated with Lut (50 μM) or AsA (50 and 75 μM) alone or in combination. (**A**) Representative images of scratches and recovery of wounded areas (marked by red lines) on cell monolayers at 0, 12 and 24 h after wounding. CaSki (**B**) and HeLa (**C**) cell semi-quantitative analysis of relative wound closure was performed by measuring the width of the wounds. Cell migration-related proteins, p-FAK, integrin β1 and paxillin, levels in CaSki (**D**) and HeLa (**E**) cells were analyzed through western blotting after 24 h of treatment. GAPDH served as the loading control. Quantitative results are shown in the lower plot. Values represent mean ± SD from three replicates. *, †, δ and γ *p* ˂ 0.05 compared with CON, L50-, A50- or A75-treated group. CON, 0.1% DMSO; L50, 50 μM luteolin; A50, 50 μM asiatic acid; A75, 75 μM asiatic acid. L50 + A50, 50 μM luteolin + 50 μM asiatic acid; L50 + A75, 50 μM luteolin + 75 μM asiatic acid.

To investigate the cell migration ability of cervical cancer cell lines treated with Lut and AsA, we performed a wound-healing assay. The cells in the untreated control group showed a faster cell migration ability than cells treated with Lut or AsA (Figure 5A–C, *p* < 0.05). Furthermore, the combination of Lut and AsA markedly inhibited the migration rate of CaSki and HeLa cells as compared with treatment with Lut or AsA alone at 12 and 24 h, although the combination with a higher dose of AsA in CaSki and HeLa cells did not. As integrin/FAK/paxillin has been reported to cause tumor migration and invasion, we next examined these modulated cell migration-related protein expressions by western blotting. Lut or AsA monotherapy significantly decreased the phospho-FAK (ratio of control: 0.69 ± 0.01 vs. 0.86 ± 0.01), integrin β1 (ratio of control: 0.94 ± 0.01 vs. 0.69 ± 0.07) and paxillin (ratio of control: 0.71 ± 0.01 vs. 0.93 ± 0.04) protein expression levels in CaSki cells, while for HeLa cells, the expressions of phospho-FAK, integrin β1 and paxillin were reduced (ratio of control: 0.90 ± 0.02 vs. 0.86 ± 0.03, 0.78 ± 0.03 vs. 0.66 ± 0.03, 0.42 ± 0.01 vs. 0.63 ± 0.02, respectively, Figure 5D,E and Figure S2, *p* < 0.05). Lut and AsA co-treatment more significantly decreased the phospho-FAK, integrin β1 and paxillin protein expressions (CaSki cells, ratio of control: 0.60 ± 0.03 vs. 0.82 ± 0.03 vs. 0.74 ± 0.02; HeLa cells, ratio of control: 0.68 ± 0.1 vs. 0.61 ± 0.03 vs. 0.26 ± 0.02, respectively). These results clearly suggested that Lut combined with AsA inhibited cancer cell migration by decreasing the integrin/FAK/paxillin signaling pathway.

### 3.6. Luteolin and Asiatic Acid Activated the Mitochondrial-Related Intrinsic Signaling Pathway

**Figure 6:**
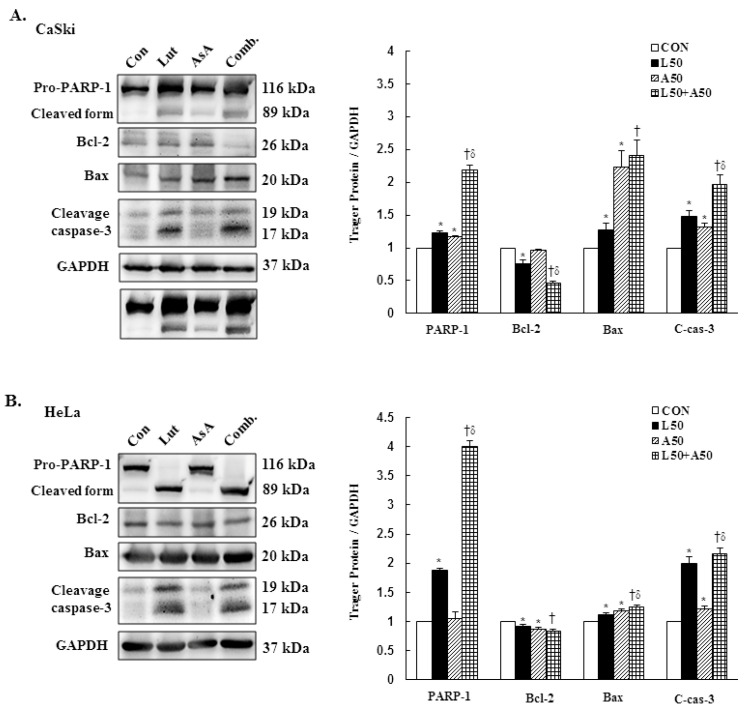
Luteolin combined with asiatic acid induced apoptosis through the mitochondrial-relative intrinsic pathway. Western blotting was used to detect the levels of apoptosis-related proteins, PARP-1, Bcl-2, Bax and caspase-3, in CaSki (**A**) and HeLa (**B**) cells treated with Lut (50 μM) or AsA (50 μM) alone or in combination for 24 h. GAPDH served as the loading control. Quantitative results are shown in the lower plot. Values represent mean ± SD from three replicates. *, † and δ *p* ˂ 0.05 compared with CON, L50- or A50-treated group. CON, 0.1% DMSO; L50, 50 μM luteolin; A50, 50 μM asiatic acid; Comb., 50 μM luteolin + 50 μM asiatic acid.

To further confirm our prior results showing that Lut and AsA treatment induced cervical cancer cell death by apoptosis, apoptosis-related proteins expressions were determined by western blotting. The western blotting findings revealed that Lut or AsA treatment significantly increased the expressions of cleaved PARP-1, pro-apoptotic protein Bax and cleaved caspase-3, whereas anti-apoptotic protein Bcl-2 was significantly decreased in expression as compared with the control in CaSki and HeLa cervical cancer cells (CaSki cells, ratio of control: 1.23 ± 0.03 vs. 1.17 ± 0.01, 1.28 ± 0.1 vs. 2.23 ± 0.25, 1.48 ± 0.09 vs. 1.32 ± 0.05, 0.76 ± 0.05 vs. 0.97 ± 0.01; HeLa cells, ratio of control: 1.88 ± 0.03 vs. 1.05 ± 0.12, 1.11 ± 0.04 vs. 1.19 ± 0.03, 1.99 ± 0.12 vs. 1.21 ± 0.06, 0.92 ± 0.04 vs. 0.87 ± 0.03, respectively). Moreover, Lut and AsA co-treatment more significantly upregulated the cleaved PARP-1, Bax and cleaved caspase-3 expressions, and downregulated the Bcl-2 expression (CaSki cells, ratio of control: 2.18 ± 0.08 vs. 2.41 ± 0.23 vs. 1.96 ± 0.15 vs. 0.47 ± 0.02; HeLa cells, ratio of control: 4.0 ± 0.1 vs. 1.25 ± 0.03 vs. 2.16 ± 0.10 vs. 0.83 ± 0.04, respectively) as compared with Lut or AsA monotherapy, although no significant differences were observed in the Bax expression in CaSki cells and Bcl-2 expression in HeLa cells compared with AsA monotherapy (Figure 6 and Figure S3, *p* < 0.05). Taken together, these findings showed that Lut and AsA induced pro-apoptotic effects in CaSki and HeLa cells through a mitochondria-related intrinsic signaling pathway.

### 3.7. Pro-Apoptotic Mechanism of Luteolin and Asiatic Acid in Cervical Cancer Cells

**Figure 7:**
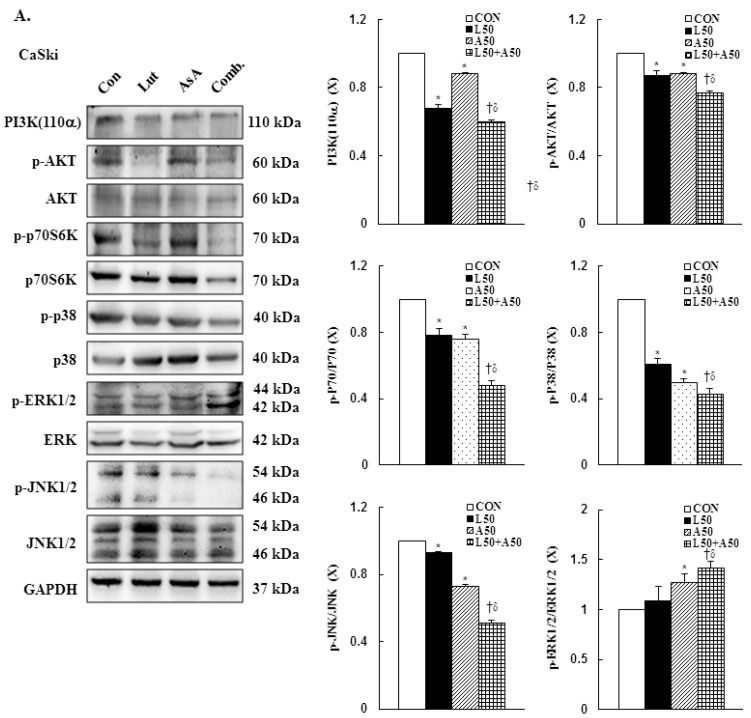

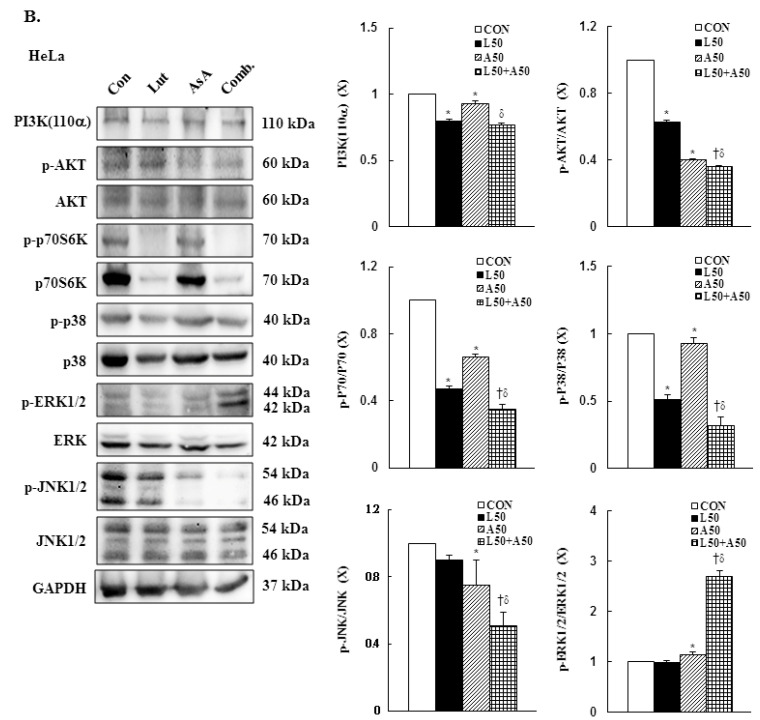
Luteolin combined with asiatic acid induced apoptosis through inhibition of PI3K/AKT and regulation of MAPKs pathways in cervical cancer cells. Western blotting was used to detect PIK3CA, p-AKT, AKT, p-70S6K, 70S6K, p-p38, p38, p-ERK1/2, ERK1/2, p-JNK1/2 and JNK1/2 levels in CaSki (**A**) and HeLa (**B**) cells treated with Lut (50 μM) or AsA (50 μM) alone or in combination for 24 h. GAPDH served as the loading control. Quantitative results are shown in the lower plot. Values represent mean ± SD from three replicates. *, † and δ *p* ˂ 0.05 compared with CON, L50- or A50-treated group. CON, 0.1% DMSO; L50, 50 μM luteolin; A50, 50 μM asiatic acid; Comb., 50 μM luteolin + 50 μM asiatic acid.

To further to understand the molecular mechanism of Lut- and AsA-induced apoptosis in cervical cancer cells, the phosphorylation statuses of PI3K(p110α), AKT, p70S6K, p38, ERK1/2 and JNK1/2 were determined by western blotting. In CaSki cells, Lut or AsA monotherapy significantly decreased phosphorylation of PI3K(p110α), AKT, p70S6K, p38 and JNK1/2, while significantly increasing phosphorylation of ERK1/2, as compared with the control (ratio of control: 0.68 ± 0.02 vs. 0.88 ± 0.01, 0.87 ± 0.03 vs. 0.88 ± 0.01, 0.78 ± 0.04 vs. 0.76 ± 0.03, 0.61 ± 0.03 vs. 0.50 ± 0.02, 0.93 ± 0.01 vs. 0.73 ± 0.01, 1.09 ± 0.14 vs. 1.27 ± 0.09, respectively). Similar results were observed for HeLa cells, with Lut or AsA monotherapy significantly decreasing PI3K(p110α), p-AKT, p-p70S6K, p-p38 and p-JNK1/2 and significantly increasing the p-ERK1/2 protein expression level (ratio of control: 0.80 ± 0.01 vs. 0.93 ± 0.02, 0.63 ± 0.01 vs. 0.40 ± 0.01, 0.47 ± 0.02 vs. 0.66 ± 0.02, 0.51 ± 0.04 vs. 0.93 ± 0.04, 0.90 ± 0.03 vs. 0.75 ± 0.15, 0.98 ± 0.04 vs. 1.14 ± 0.05, respectively), although no significant difference were observed the p-JNK1/2 expression (ratio of control: 0.90 ± 0.03) in HeLa cells and p-ERK1/2 expression in both CaSki and HeLa cells after Lut monotherapy (ratio of control: 1.09 ± 0.14 vs. 0.98 ± 0.04, respectively; Figure 7, Figures S4 and S5, *p* < 0.05). Moreover, combination treatment with Lut and AsA more significantly decreased the PI3K(p110α), p-AKT, p-p70S6K, p-p38 and p-JNK1/2 expressions, and more synergistically increased the p-ERK1/2 expression level in both CaSki and HeLa cells (CaSki cells, ratio of control: 0.60 ± 0.01 vs. 0.77 ± 0.01 vs. 0.48 ± 0.03 vs. 0.43 ± 0.03 vs. 0.51 ± 0.02 vs. 1.42 ± 0.06; HeLa cells, ratio of control: 0.77 ± 0.01 vs. 0.36 ± 0.01 vs. 0.35 ± 0.03 vs. 0.32 ± 0.06 vs. 0.51 ± 0.08 vs. 2.69 ± 0.13, respectively), as compared with Lut or AsA alone. These results confirmed that Lut- and AsA-induced apoptosis in cervical cancer cells might involve the PI3K/AKT/p70S6K and p38/ERK1/2/JNK1/2 pathways.

### 3.8. Caspase-3-Mediated Intrinsic Apoptosis Pathway Is Involved in Luteolin- and Asiatic Acid-Induced Anti-Proliferation in Cervical Cancer Cells

**Figure 8:**
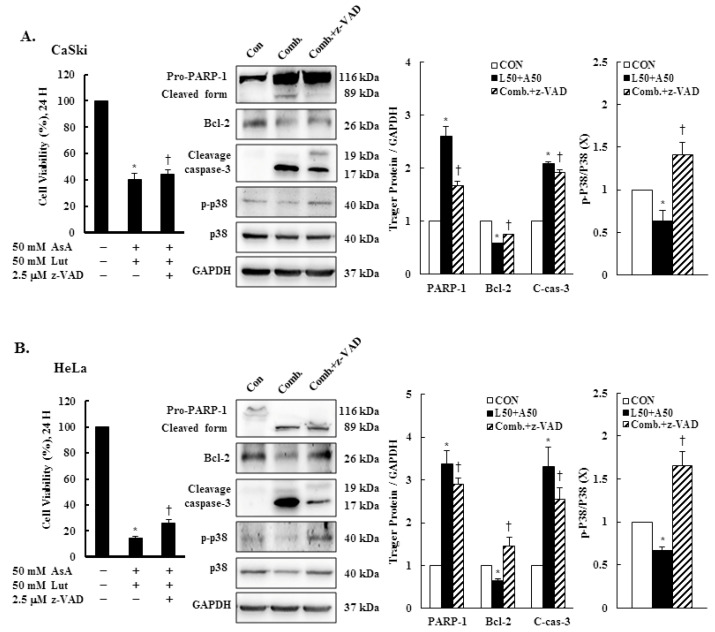
z-VAD-fmk altered apoptosis-related protein expressions. CaSki and HeLa cells were pretreated with z-VAD-fmk (2.5 μM) for 2 h and then treated with or without Lut (50 μM) and AsA (50 μM) for 24 h. CaSki (**A**) and HeLa (**B**) cell viabilities were examined using a CCK-8 assay. The expressions of PARP-1, Bcl-2, caspase-3, p-p38 and p38 were detected through western blotting. GAPDH served as the loading control. Quantitative results are shown in the lower plot. Values represent mean ± SD from three replicates. * and † *p* ˂ 0.05 compared with CON or AsA + Lut-treated group. CON, 0.1% DMSO; Comb., 50 μM luteolin + 50 μM asiatic acid; Comb. + z-VAD, 50 μM luteolin + 50 μM asiatic acid + 2.5 μM z-VAD-fmk.

To further confirm the involvement of a caspase-dependent apoptotic pathway, CaSki and HeLa cells were pretreated with pan-caspase inhibitor z-VAD-fmk (2.5 μM) for 2 h, followed by combined treatment with Lut and AsA. The results showed that z-VAD-fmk significantly reduced the anti-proliferation effects of Lut and AsA on cervical cancer cells (CaSki cells, 40.7 ± 4.1% vs. 44.1 ± 3.9%; HeLa cells, 14.8 ± 1.3% vs. 25.7 ± 2.9%). The western blot findings revealed that co-treated cells had significantly lower expressions of cleaved PARP-1 and cleaved caspase-3 proteins (CaSki cells, ratio of control: 2.60 ± 0.19 vs. 1.67 ± 0.08, 2.08 ± 0.04 vs. 1.91 ± 0.06; HeLa cells, ratio of control: 3.38 ± 0.03 vs. 2.90 ± 0.14, 3.31 ± 0.45 vs. 2.55 ± 0.27, respectively) and higher expressions of Bcl-2 and p-p38 proteins (CaSki cells, ratio of control: 0.58 ± 0.01 vs. 0.75 ± 0.01, 0.63 ± 0.13 vs. 1.41 ± 0.14; HeLa cells, ratio of control: 0.65 ± 0.04 vs. 1.46 ± 0.19, 0.67 ± 0.04 vs. 1.65 ± 0.17, respectively), as compared with cells treated with Lut and AsA in combination (Figure 8 and Figure S6, *p* < 0.05). These results clearly indicated that the anti-proliferation effect of Lut combined with AsA in cervical cancer cells acted through a caspase-3-mediated intrinsic apoptosis pathway.

### 3.9. Luteolin and Asiatic Acid Suppressed Cervical Cancer Cell-Derived Xenograft Tumors

**Figure 9:**
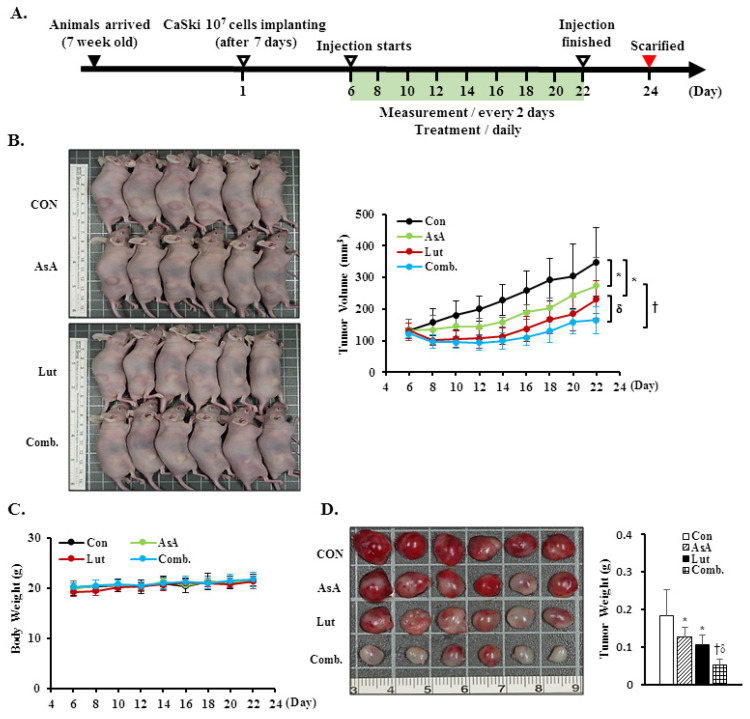

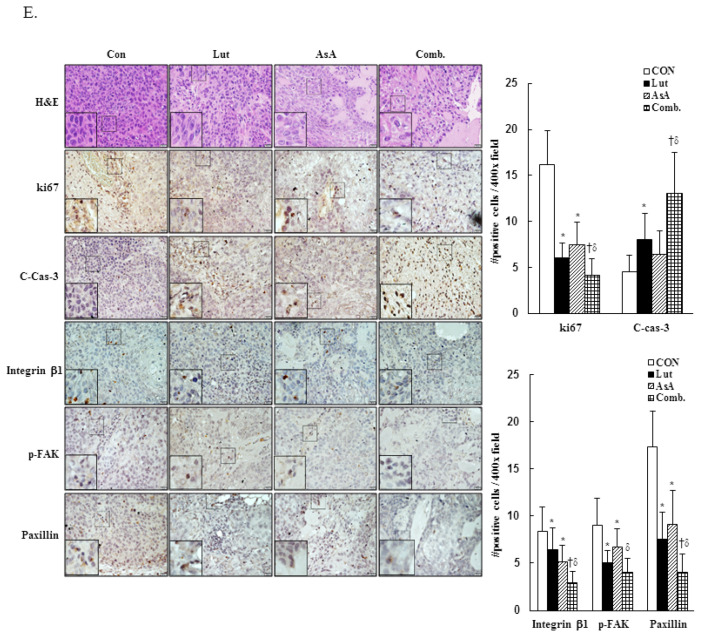
Luteolin combined with asiatic acid suppressed tumor growth and tumor weight increase in BALB/c nude mice. CaSki cells (1 × 10^7^ cells) were injected subcutaneously into the right flank of nude mice. Treatment was initiated 6 days after CaSki cell injection; Lut (50 mg/kg) or AsA (100 mg/kg), in combination or vehicle control alone (10% DMSO) was injected intraperitoneally every day. (**A**) Schematic representation of the experiment. (**B**) Representative images of tumors. (**C**) Tumor volume and body weight were measured every two days. (**D**) Tumor weight was recorded after sacrifice on day 22. (**E**) Representative histological sections of the tumor tissue were stained with hematoxylin and eosin, ki67, caspase-3, integrin β1, p-FAK and paxillin (shown as brown staining; H&E, 200x, bar = 50 μm; IHC, 400×, bar = 20 μm). Quantitative results are shown in the lower plot. Values represent mean ± SD (*n* = 6/group). *, † and δ *p* < 0.05 compared with CON, Lut- or AsA-treated groups. CON, control; Lut, luteolin; AsA, asiatic acid; Comb., luteolin combined with asiatic acid.

To further confirm the in vitro anti-proliferation effects of Lut and AsA, a CaSki xenograft tumor model was constructed in BALB/c nude mice. The schematic timeline of this experiment was as shown in Figure 9A. Lut or AsA markedly inhibited the in vivo tumor growth of CaSki xenografts, particularly Lut combined with AsA. After treatment for 16 days, the average tumor volume of the CaSki xenograft tumors was 349.4 ± 108.7 mm^3^ vs. 274.1 ± 88.2 mm^3^ vs. 230.3 ± 58.4 mm^3^ vs. 165.7 ± 42.6 mm^3^ in the vehicle control, AsA-, Lut- and Lut plus AsA-treated groups, while the tumor growth inhibition rate was 21.6% vs. 34.1% vs. 52.6%, respectively (Figure 9B, *p* < 0.05). No significant difference in body weight between groups was observed during the experimental period (21.3 ± 1.4 g vs. 21.4 ± 1.2 g vs. 21.4 ± 0.9 g vs. 21.7 ± 1.5 g, respectively) and the experimental animal survival rate was 100%, suggesting that treatment with Lut and AsA alone or in combination did not cause host drug toxicity (Figure 9C). The average tumor weight was significantly reduced in the AsA-, Lut- and Lut plus AsA-treated groups (0.13 ± 0.03 g vs. 0.11 ± 0.03 g vs. 0.05 ± 0.02 g, respectively) as compared with the control group (0.18 ± 0.07 g, Figure 9D, *p* < 0.05). Moreover, in performing H&E staining, we found that the Lut plus AsA treatment more effectively reduced the tumor cellularity and apoptosis in the CaSki xenografts than AsA or Lut monotherapy. Similarly, consistent with the in vitro findings in this study, IHC study demonstrated more significantly downregulated expressions of ki67 (4.1 ± 1.8 positive cells), integrin β1 (2.9 ± 1.2 positive cells), p-FAK (4.0 ± 1.5 positive cells) and paxillin (4.0 ± 2.0 positive cells) and an upregulated cleaved caspase-3 (13.0 ± 4.5 positive cells) protein expression in tumors treated with Lut plus AsA as compared with the AsA-, Lut- and control- groups (ki67, 7.5 ± 2.4 vs. 6.0 ± 1.6 vs. 16.2 ± 3.7 positive cells; integrin β1, 5.1 ± 1.8 vs. 6.4 ± 2.3 vs. 8.4 ± 2.6 positive cells; p-FAK, 6.7 ± 1.9 vs. 5.0 ± 1.3 vs. 9.0 ± 2.9 positive cells; paxillin, 9.1 ± 3.6 vs. 7.5 ± 2.9 vs. 17.3 ± 3.8 positive cells; cleaved caspase-3, 6.4 ± 2.6 vs. 8.0 ± 2.9 vs. 4.5 ± 1.8 positive cells, respectively; Figure 9E, *p* < 0.05). These results further suggested that Lut plus AsA combined treatment acted as an anti-proliferative, anti-migratory and pro-apoptotic agent in vivo.

## 4. Discussion

Cervical cancer remains a leading cause of high mortality and morbidity in women worldwide. Although chemotherapy can effectively to improve the survival of cervical cancer patients, it is also prone to increase drug resistance, toxic side effect and complications, causing great suffering to patients. Therefore, a new oncology therapy for cervical cancer by plant-derived natural products (such as alkaloid, flavonoids, phenols and terpenoids) has attracted attention. AsA and Lut themselves are rich in natural triterpenoids and flavonoids, and their therapeutic effects on multiple cancers’ proliferation and metastasis have been studied. Based on this, this study aimed to assess the anticancer effect of AsA and Lut in human cervical cancer. Simultaneously, we also evaluated whether Lut combined with AsA more notably enhanced the anticancer effect than Lut or AsA alone in vitro and in vivo. Our study results showed that AsA or Lut alone can reduce the cell viability of human cervical cancer cells (CaSki, HeLa and C33A, Figure 1), which were consistent with previous studies showing that AsA significantly inhibited cell growth of ovarian cancer [19], nasopharyngeal carcinoma [22] and breast cancer [23,24]; and Lut showed results against leukemia [48], prostate cancer [49] and breast cancer [41,42]. In addition, the Lut combined with AsA treatment also had a significantly more synergistic effect against cervical cancer growth than Lut or AsA alone in CaSki and HeLa cells, and synergistically inhibited xenograft animal tumor growth (Figure 9). These results were consistent with those previously reported by Jeon et al. and Johnson et al., who indicated that the combined use of Lut and paclitaxel or gemcitabine synergistically augmented anticancer activity in breast and pancreatic cancer treatment [50,51]. Lian et al. also showed that AsA combined with naringenin could have a greater effect to suppress melanoma and lung carcinoma growth [27]. In addition, Ham et al. revealed that Lut treatment resulted in a more significant cytotoxic effect in HPV-positive cervical cancer cells and had less effect on HPV-negative cervical cancer C33A cells [52], with as Kim et al. indicating that bee venom could significantly promote the suppression of cell growth in HPV16-infected cells (CaSki cells) and HPV18-infected cells (HeLa cells) compared to HPV-noninfected cells (C33A cells), via downregulation of HPV E6/E7 expression [53]. Similarly, AsA, Lut and combination treatment promoted cell apoptosis (as upregulation of Bax/Bcl-2 ratio, cleaved-PARP-1 and cleaved caspase-3) and inhibited cell migration (downregulation of integrin β1, FAK and paxillin) in both CaSki and HeLa cells and xenograft animal model (Figure 2, Figure 5, Figure 6 and Figure 9), which were consistent results of previous studies. [22,39,54,55].

As consistent with results of previous studies, AsA induced cell death by causing cancer cells to undergo G0/G1- (SKOV3 and OVCAR-3) [19 Ren 2016] or S-G2/M-phases arrest (MCF-7, MDA-MB-231, SW480 and HCT116) [21,56] and Lut was involved in G0/G1- (HepG2, NCI-H1975 and NCI-H1650) [57,58] or G2/M- phases arrest (A549, HeLa, LoVo, HCT-116 and HT-29) [44,59,60,61]. Lut combined with oxaliplatin treatment promoted SGC-7901 cell apoptosis by altering G0/G1 phase proportion [62]. In our study, AsA, Lut and combination treatment were causing sub-G1 phase arrest in CaSki and HeLa cells (Figure 3), which was consistent with Horninaka et al., who revealed that Lut combined with TRAIL treatment synergistically induced cell cycle sub-G1 arrest in HeLa cells [46]. These differences in results represented in the cell cycle phase arrest of AsA and Lut treatment may be associated with cancer cell type specificity and individual AsA and Lut bioavailability (dose- or time-stimuli manner). Payent et al., [63] and Lv et al. [15] revealed that mitochondrial ROS exerted dual roles in tumor-suppression or tumor-promotion, and in the presence of a high level of mitROS that will cause tissue damage and cell death, AsA treatment could remarkably inhibit ROS formation and increase the GSH, superoxide dismutase (SOD) and CAT levels to attenuate oxidative stress damage, whereas Park et al. [64] and Li et al. [28] indicated that AsA caused the induction of ROS generation to induce apoptosis in SK-MEL-2 and HepG2 cells. Our results showed that AsA, Lut and combination significantly increased mitROS production and promoted cancer cell apoptosis (Figure 2 and Figure 4), and, as Imhoff et al. [65] revealed, high levels of ROS-induced mitROS will cause cell apoptosis and autophagy to reduce tumorigenesis, thereby affecting the GSH and CAT expressions, which have not increased. However, luteolin was be found to increase or attenuate productions of ROS in HepG2 and MCF-7 cells, respectively [66,67].

Previous research revealed that the Lut or AsA induced apoptosis through the inactivation of the PI3K/AKT/mTOR/p70S6K pathway in some common cancers, including glioblastoma [68], lung [69], ovarian [19], colon [21] and breast cancers [41,70], which is consistent with our study finding that AsA, Lut and combination treatment inhibited PI3K, p-AKT and p-p70S6K protein expressions in CaSki and HeLa cells (Figure 7). However, Zhou et al. [71] showed that Lut activity against prostate cancer PC3 cell invasion was through active AKT to inhibit mdm2 expression, resulting in attenuated E-cadherin expression. In addition, activation of p-p38 and p-JNK1/2 were inhibited with AsA, Lut and combination treatment in CaSki and HeLa cells, whereas p-ERK1/2 was not inhibited. Caspase inhibitor z-VAD-fmk significantly reversed CaSki and C33A cell growth and increased p-p38 activation in cells co-treated with Lut and AsA (Figure 8), which can be explained by the fact that p38 regulating Lut combined with AsA-induced cell apoptosis. These findings were consistent with those of previous reports that Lut and celecoxib combinatorial therapy protected against breast cancer cells (MDA-MB-231 and SkBr3) through activating ERK and inactivating AKT and through inactivating ERK and AKT in other breast cancer cells (MCF-7 and MCF7/HER18) [72]. However, Potočnjak et al. showed the Lut increased colon cancer SW620 cells’ viability by upregulation of the p-ERK/p-JNK/p-p38 pathway [43]. Hsu et al. [56] indicated that AsA induced apoptosis and cell cycle arrest through activating the ERK/p38 MAPK pathway in breast cancers (MCF-7 and MDA-MB-231). Hish et al. and Chen et al. also showed that AsA suppressed human renal cancer cell migration and invasion via inhibition of the p-ERK/p-p38MAPK pathway [26], while AsA-induced apoptosis in cisplatin-resistant nasopharyngeal carcinoma cells was by activation of the p-JNK/p-p38MAPK pathway and inactivation of the p-ERK pathway [22]. These inconsistent results of the Lut- or AsA-mediated signaling pathway may be dependent on cell type and dose- or time-stimuli manner. The detailed mechanism will require further clarification.

## 5. Conclusions

**Figure 10:**
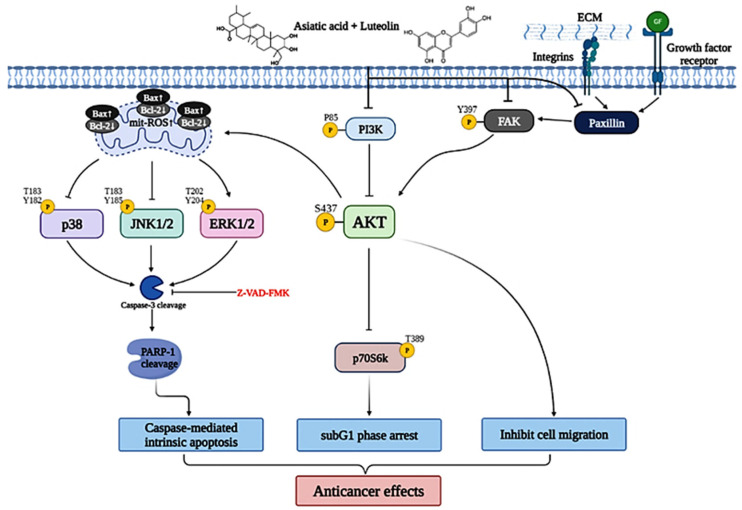
Schematic diagram of luteolin combined with asiatic acid’s anticancer molecular mechanism in cervical cancer. Lut combined with AsA mainly modulates FAK signaling (integrin β1, paxillin and FAK) and PI3K/AKT signaling (PI3K, AKT and p70S6K) and causes JNK/p38 downregulation and ERK upregulation, inactivating or activating various signaling targets, such as Bcl2, Bax, mitROS, caspase-3 and RARP1; this leads to the induction of caspase-mediated intrinsic apoptosis, sub-G1 phase arrest and inhibition of cancer cell migration, which increase the anticancer effect on cervical cancer.

Drug resistance and side effects are frequently developed in patients undergoing cervical cancer chemotherapy. A novel anticancer agent, especially derived from natural sources, is required to address these issues. Our study is the first to have demonstrated an anticancer effect of combined Lut plus AsA treatment on cervical cancer in vitro and in vivo. Lut combined with AsA effectively reduced cervical cancer cell viability by arresting the cell cycle in the sub-G1 phase and inducing caspase-mediated intrinsic apoptosis. Lut combined with AsA treatment downregulated PI3K/AKT signaling (PI3K, AKT and p70S6K), JNK/p38 MAPK signaling and FAK signaling (integrin β1, paxillin and FAK) and upregulated ERK signaling to induce apoptosis and inhibit cancer cell migration, resulting in an anticancer effect on cervical cancer (Figure 10). Furthermore, our in vivo study revealed that Lut combined with AsA markedly inhibited cervical cancer cell-derived xenograft tumor growth, and the cytotoxic effect of Lut combined with AsA was minimal or nonexistent. Collectively, the present study showed that Lut combined with AsA may be used as an anticancer agent to improve the prognosis of cervical cancer. Indeed, with additional research to develop standardized dosages, Lut and AsA combination therapy could also be applied in clinical medicine.

## Data Availability

The datasets generated and/or analyzed during the current study are available from the corresponding author on reasonable request.

## Supplementary Materials

The following are available online at https://www.mdpi.com/article/10.3390/cancers15020548/s1, Figure S1: Luteolin and asiatic acid inhibit cell proliferation in cervical cancer cell lines for 24, 48 and 72 h. Figure S2: Luteolin and asiatic acid inactivate the integrin β1/FAK/paxillin pathway. Figure S3: Luteolin and asiatic acid activate the mitochondrial-related intrinsic pathway (PARP-1, Bcl-2, Bax and caspase-3). Figure S4: Luteolin and asiatic acid downregulated PI3K/AKT/p70S6K pathway. Figure S5: Luteolin and asiatic acid modulated MAPKs (p38, ERK and JNK) pathway. Figure S6: Caspase-3 and p38 involves luteolin- and asiatic acid-induced apoptosis.

## References

[1] Sung H., Ferlay J., Siegel R.L., Laversanne M., Soerjomataram I., Jemal A., Bray F. (2021). Global cancer statistics 2020: GLO-BOCAN estimates of incidence and mortality worldwide for 36 cancers in 185 countries. CA Cancer J. Clin..

[2] Bethesda SEER Cancer Stat Facts: Cervical Cancer. National Cancer Institute. https://seer.cancer.gov/statfacts/html/cervix.html.

[3] Okunade K.S. (2020). Human papillomavirus and cervical cancer. J. Obstet. Gynaecol..

[4] de Sanjose S., Quint W.G.V., Alemany L., Geraets D.T., Klaustermeier J.E., Lloveras B., Tous S., Felix A., Bravo L.E., Shin H.-R. (2010). Human papillomavirus genotype attribution in invasive cervical cancer: A retrospective cross-sectional worldwide study. Lancet Oncol..

[5] Geraets D., Alemany L., Guimera N. (2012). RIS HPV TT study group. Detection of rare and possibly carcinogenic human papil-lomavirus genotypes as single infections in invasive cervical cancer. J. Pathol..

[6] Ouyang L., Luo Y., Tian M., Zhang S.-Y., Lu R., Wang J.-H., Kasimu R., Li X. (2014). Plant natural products: From traditional compounds to new emerging drugs in cancer therapy. Cell Prolif..

[7] Aung T.N., Qu Z., Kortschak R.D., Adelson D.L. (2017). Understanding the Effectiveness of Natural Compound Mixtures in Cancer through Their Molecular Mode of Action. Int. J. Mol. Sci..

[8] Kikuchi H., Yuan B., Hu X., Okazaki M. (2019). Chemopreventive and anticancer activity of flavonoids and its possibility for clinical use by combining with conventional chemotherapeutic agents. Am. J. Cancer Res..

[9] Lv J., Sharma A., Zhang T., Wu Y., Ding X. (2018). Pharmacological review on asiatic acid and its derivatives: A potential com-pound. SLAS Technol..

[10] Bunbupha S., Pakdeechote P., Kukongviriyapan U., Prachaney P., Kukongviriyapan V. (2014). Asiatic Acid Reduces Blood Pres-sure by Enhancing Nitric Oxide Bioavailability with Modulation of eNOS and p47ᵖʰᵒˣ Expression in l-NAME-induced Hyper-tensive Rats. Phytother. Res..

[11] Jiang W., Li M., He F., Bian Z., He Q., Wang X., Yao W., Zhu L. (2016). Neuroprotective effect of asiatic acid against spinal cord injury in rats. Life Sci..

[12] Jew S.-S., Yoo C.-H., Lim D.-Y., Kim H., Mook-Jung I., Jung M.W., Choi H., Jung Y.-H., Kim H., Park H.-G. (2000). Structure–activity relationship study of asiatic acid derivatives against beta amyloid (Aβ)-induced neurotoxicity. Bioorganic Med. Chem. Lett..

[13] Ramachandran V., Saravanan R. (2015). Glucose uptake through translocation and activation of GLUT4 in PI3K/Akt signaling pathway by asiatic acid in diabetic rats. Hum. Exp. Toxicol..

[14] Yang C., Guo Y., Huang T.-S., Zhao J., Huang X.-J., Tang H.-X., An N., Pan Q., Xu Y.-Z., Liu H.-F. (2018). Asiatic acid protects against cisplatin-induced acute kidney injury via anti-apoptosis and anti-inflammation. Biomed. Pharmacother..

[15] Lv H., Qi Z., Wang S., Feng H., Deng X., Ci X. (2017). Asiatic Acid Exhibits Anti-inflammatory and Antioxidant Activities against Lipopolysaccharide and d-Galactosamine-Induced Fulminant Hepatic Failure. Front. Immunol..

[16] Liu W.-H., Liu T.-C., Mong M.-C. (2015). Antibacterial effects and action modes of asiatic acid. Biomed. Pharmacother..

[17] Somboonwong J., Kankaisre M., Tantisira B., Tantisira M.H. (2012). Wound healing activities of different extracts of *Centella asiatica* in incision and burn wound models: An experimental animal study. BMC Complement. Altern. Med..

[18] Dong M., Zeng J., Yang C., Qiu Y., Wang X. (2022). Asiatic Acid Attenuates Osteoporotic Bone Loss in Ovariectomized Mice Through Inhibiting NF-kappaB/MAPK/Protein Kinase B Signaling Pathway. Front. Pharmacol..

[19] Ren L., Cao Q.-X., Zhai F.-R., Yang S.-Q., Zhang H.-X. (2016). Asiatic acid exerts anticancer potential in human ovarian cancer cells via suppression of PI3K/Akt/mTOR signalling. Pharm. Biol..

[20] Wu T., Geng J., Guo W., Gao J., Zhu X. (2016). Asiatic acid inhibits lung cancer cell growth in vitro and in vivo by destroying mitochondria. Acta Pharm. Sin. B.

[21] Hao Y., Huang J., Ma Y., Chen W., Fan Q., Sun X., Shao M., Cai H. (2018). Asiatic acid inhibits proliferation, migration and induces apoptosis by regulating Pdcd4 via the PI3K/Akt/mTOR/p70S6K signaling pathway in human colon carcinoma cells. Oncol. Lett..

[22] Liu Y.-T., Chuang Y.-C., Lo Y.-S., Lin C.-C., Hsi Y.-T., Hsieh M.-J., Chen M.-K. (2020). Asiatic acid, extracted from *Centella asiatica* and induces apoptosis pathway through the phosphorylation p38 mitogen-activated protein kinase in cisplatin-resistant na-sopharyngeal carcinoma cells. Biomolecules.

[23] Tian M., Chen K., Huang J., Chu D., Li J., Huang K., Ma C. (2021). Asiatic acid inhibits angiogenesis and vascular permeability through the VEGF/VEGFR2 signaling pathway to inhibit the growth and metastasis of breast cancer in mice. Phytotherapy Res..

[24] Zhu Z., Cui L., Yang J., Vong C.T., Hu Y., Xiao J., Chan G., He Z., Zhong Z. (2021). Anticancer effects of asiatic acid against doxorubicin-resistant breast cancer cells via an AMPK-dependent pathway in vitro. Phytomedicine.

[25] Yan B., Chen X., Liu J., Liu S., Zhang J., Zeng Q., Duan J. (2021). Asiatic Acid Induces Mitochondrial Apoptosis via Inhibition of JAK2/STAT3 Signalling Pathway in Human Osteosarcoma. Folia Biol..

[26] Huang C.-F., Hung T.-W., Yang S.-F., Tsai Y.-L., Yang J.-T., Lin C., Hsieh Y.-H. (2022). Asiatic acid from centella asiatica exert anti-invasive ability in human renal cancer cells by modulation of ERK/p38MAPK-mediated MMP15 expression. Phytomedicine.

[27] Lian G.-Y., Wang Q.-M., Tang P.M.-K., Zhou S., Huang X.-R., Lan H.-Y. (2018). Combination of asiatic acid and naringenin modulates NK cell anti-cancer immunity by rebalancing Smad3/Smad7 signaling. Mol. Ther..

[28] Li J.-F., Huang R.-Z., Yao G.-Y., Ye M.-Y., Wang H.-S., Pan Y.-M., Xiao J.-T. (2014). Synthesis and biological evaluation of novel aniline-derived asiatic acid derivatives as potential anticancer agents. Eur. J. Med. Chem..

[29] Jing Y., Wang G., Ge Y., Xu M., Gong Z. (2015). Synthesis, Anti-Tumor and Anti-Angiogenic Activity Evaluations of Asiatic Acid Amino Acid Derivatives. Molecules.

[30] Gonçalves B.M., Salvador J.A., Marín S., Cascante M. (2016). Synthesis and anticancer activity of novel fluorinated asiatic acid derivatives. Eur. J. Med. Chem..

[31] Chen Z., Kong S., Song F., Li L., Jiang H. (2012). Pharmacokinetic study of luteolin, apigenin, chrysoeriol and diosmetin after oral administration of Flos Chrysanthemi extract in rats. Fitoterapia.

[32] Lim S.H., Jung S.K., Byun S., Lee E.J., Hwang J.A., Seo S.G., Kim Y.A., Yu J.G., Lee K.W., Lee H.J. (2013). Luteolin suppresses UVB-induced photoageing by targeting JNK1 and p90^RSK2^. J. Cell. Mol. Med..

[33] Lin Y., Shi R., Wang X., Shen H.-M. (2008). Luteolin, a Flavonoid with Potential for Cancer Prevention and Therapy. Curr. Cancer Drug Targets.

[34] Aziz N., Kim M.-Y., Cho J.Y. (2018). Anti-inflammatory effects of luteolin: A review of in vitro, in vivo, and in silico studies. J. Ethnopharmacol..

[35] Seelinger G., Merfort I., Schempp C.M. (2008). Anti-Oxidant, Anti-Inflammatory and Anti-Allergic Activities of Luteolin. Planta Medica.

[36] Cassidy A., Minihane A.M. (2017). The role of metabolism (and the microbiome) in defining the clinical efficacy of dietary flavonoids. Am. J. Clin. Nutr..

[37] Birt D.F., Hendrich S., Wang W. (2001). Dietary agents in cancer prevention: Flavonoids and isoflavonoids. Pharmacol. Ther..

[38] Martin K.R. (2006). Targeting Apoptosis with Dietary Bioactive Agents. Exp. Biol. Med..

[39] Imran M., Rauf A., Abu-Izneid T., Nadeem M., Shariati M.A., Khan I.A., Imran A., Orhan I.E., Rizwan M., Atif M. (2019). Luteolin, a flavonoid, as an anticancer agent: A review. Biomed. Pharmacother..

[40] Ganai S.A., Sheikh F.A., Baba Z.A., Mir M.A., Mantoo M.A., Yatoo M.A. (2021). Anticancer activity of the plant flavonoid luteolin against preclinical models of various cancers and insights on different signalling mechanisms modulated. Phytother. Res..

[41] Wu H.-T., Lin J., Liu Y.-E., Chen H.-F., Hsu K.-W., Lin S.-H., Peng K.-Y., Lin K.-J., Hsieh C.-C., Chen D.-R. (2020). Luteolin suppresses androgen receptor-positive triple-negative breast cancer cell proliferation and metastasis by epigenetic regulation of MMP9 expression via the AKT/mTOR signaling pathway. Phytomedicine.

[42] Tsai K.-J., Tsai H.-Y., Tsai C.-C., Chen T.-Y., Hsieh T.-H., Chen C.-L., Mbuyisa L., Huang Y.-B., Lin M.-W. (2021). Luteolin Inhibits Breast Cancer Stemness and Enhances Chemosensitivity through the Nrf2-Mediated Pathway. Molecules.

[43] Potočnjak I., Šimić L., Gobin I., Vukelić I., Domitrović R. (2020). Antitumor activity of luteolin in human colon cancer SW620 cells is mediated by the ERK/FOXO3a signaling pathway. Toxicol. Vitr..

[44] Krifa M., Alhosin M., Muller C.D., Gies J.-P., Chekir-Ghedira L., Ghedira K., Mély Y., Bronner C., Mousli M. (2013). *Limoniastrum guyonianum* aqueous gall extract induces apoptosis in human cervical cancer cells involving p16INK4A re-expression related to UHRF1 and DNMT1 down-regulation. J. Exp. Clin. Cancer Res..

[45] Shi R.-X., Ong C.-N., Shen H.-M. (2004). Luteolin sensitizes tumor necrosis factor-α-induced apoptosis in human tumor cells. Oncogene.

[46] Horinaka M., Yoshida T., Shiraishi T., Nakata S., Wakada M., Nakanishi R., Nishino H., Sakai T. (2005). The combination of TRAIL and luteolin enhances apoptosis in human cervical cancer HeLa cells. Biochem. Biophys. Res. Commun..

[47] Chou T.C., Talalay P. (1984). Quantitative analysis of dose-effect relationships: The combined effects of multiple drugs or enzyme inhibitors. Adv. Enzym. Regul..

[48] Cheng A.-C., Huang T.-C., Lai C.-S., Pan M.-H. (2005). Induction of apoptosis by luteolin through cleavage of Bcl-2 family in human leukemia HL-60 cells. Eur. J. Pharmacol..

[49] Chiu F.-L., Lin J.-K. (2007). Downregulation of androgen receptor expression by luteolin causes inhibition of cell proliferation and induction of apoptosis in human prostate cancer cells and xenografts. Prostate.

[50] Jeon Y.-W., Suh Y.J. (2012). Synergistic apoptotic effect of celecoxib and luteolin on breast cancer cells. Oncol. Rep..

[51] Johnson J.L., Dia V.P., Wallig M., de Mejia E.G. (2015). Luteolin and Gemcitabine Protect Against Pancreatic Cancer in an Orthotopic Mouse Model. Pancreas.

[52] Ham S., Kim K.H., Kwon T.H., Bak Y., Lee D.H., Song Y.S., Park S.-H., Park Y.S., Kim M.S., Kang J.W. (2014). Luteolin induces intrinsic apoptosis via inhibition of E6/E7 oncogenes and activation of extrinsic and intrinsic signaling pathways in HPV-18-associated cells. Oncol. Rep..

[53] Kim Y.-W., Chaturvedi P.K., Chun S.N., Lee Y.G., Ahn W.S. (2015). Honeybee venom possesses anticancer and antiviral effects by differential inhibition of HPV E6 and E7 expression on cervical cancer cell line. Oncol. Rep..

[54] Wu Q., Lv T., Chen Y., Wen L., Zhang J., Jiang X., Liu F. (2012). Apoptosis of HL-60 human leukemia cells induced by Asiatic acid through modulation of B-cell lymphoma 2 family proteins and the mitogen-activated protein kinase signaling pathway. Mol. Med. Rep..

[55] Choi H.-J., Choi H.-J., Chung T.-W., Ha K.-T. (2016). Luteolin inhibits recruitment of monocytes and migration of Lewis lung car-cinoma cells by suppressing chemokine (C–C motif) ligand 2 expression in tumor-associated macrophage. Biochem. Biophys. Res. Commun..

[56] Hsu Y.-L., Kuo P.-L., Lin L.-T., Lin C.-C. (2004). Asiatic Acid, a Triterpene, Induces Apoptosis and Cell Cycle Arrest through Activation of Extracellular Signal-Regulated Kinase and p38 Mitogen-Activated Protein Kinase Pathways in Human Breast Cancer Cells. J. Pharmacol. Exp. Ther..

[57] Yee S.B., Choi H.J., Chung S.W., Park D.H., Sung B., Chung H.Y., Kim N.D. (2015). Growth inhibition of luteolin on HepG2 cells is induced via p53 and Fas/Fas-ligand besides the TGF-β pathway. Int. J. Oncol..

[58] Zhang M., Wang R., Tian J., Song M., Zhao R., Liu K., Zhu F., Shim J.H., Dong Z., Lee M.H. (2021). Targeting LIMK1 with luteolin inhibits the growth of lung cancer in vitro and in vivo. J. Cell. Mol. Med..

[59] Cai X., Ye T., Liu C., Lu W., Lu M., Zhang J., Wang M., Cao P. (2011). Luteolin induced G2 phase cell cycle arrest and apoptosis on non-small cell lung cancer cells. Toxicol. Vitr..

[60] Chen Z., Zhang B., Gao F., Shi R. (2018). Modulation of G2/M cell cycle arrest and apoptosis by luteolin in human colon cancer cells and xenografts. Oncol. Lett..

[61] Song Y., Yu J., Li L., Wang L., Dong L., Xi G., Lu Y.J., Li Z. (2022). Luteolin impacts deoxyribonucleic acid repair by modulating the mitogen-activated protein kinase pathway in colorectal cancer. Bioengineered.

[62] Ren L.-Q., Li Q., Zhang Y. (2020). Luteolin Suppresses the Proliferation of Gastric Cancer Cells and Acts in Synergy with Oxaliplatin. BioMed Res. Int..

[63] Payen V.L., Zampieri L.X., Porporato P.E., Sonveaux P. (2019). Pro- and antitumor effects of mitochondrial reactive oxygen species. Cancer Metastasis Rev..

[64] Park B.C., Bosire K.O., Lee E.-S., Lee Y.S., Kim J.-A. (2005). Asiatic acid induces apoptosis in SK-MEL-2 human melanoma cells. Cancer Lett..

[65] Imhoff B.R., Hansen J.M. (2009). Extracellular redox status regulates Nrf2 activation through mitochondrial reactive oxygen species. Biochem. J..

[66] Hwang J.-T., Park O.J., Lee Y.K., Sung M.J., Hur H.J., Kim M.S., Ha J.H., Kwon D.Y. (2011). Anti-tumor effect of luteolin is accompanied by AMP-activated protein kinase and nuclear factor-κB modulation in HepG2 hepatocarcinoma cells. Int. J. Mol. Med..

[67] Sato Y., Sasaki N., Saito M., Endo N., Kugawa F., Ueno A. (2015). Luteolin Attenuates Doxorubicin-Induced Cytotoxicity to MCF-7 Human Breast Cancer Cells. Biol. Pharm. Bull..

[68] Cheng W.-Y., Chiao M.-T., Liang Y.-J., Yang Y.-C., Shen C.-C., Yang C.-Y. (2013). Luteolin inhibits migration of human glioblas-toma U-87 MG and T98G cells through downregulation of Cdc42 expression and PI3K/AKT activity. Mol. Biol. Rep..

[69] Chen K.-C., Chen C.-Y., Lin C.-J., Yang T.-Y., Chen T.-H., Wu L.-C., Wu C.-C. (2013). Luteolin attenuates TGF-β1-induced epi-thelial–mesenchymal transition of lung cancer cells by interfering in the PI3K/Akt–NF-κB–Snail pathway. Life Sci..

[70] Gou X.-J., Bai H.-H., Liu L.-W., Chen H.-Y., Shi Q., Chang L.-S., Ding M.-M., Zhou M.-X., Chen W.-L., Zhang L.-M. (2020). Asiatic Acid Interferes with Invasion and Proliferation of Breast Cancer Cells by Inhibiting WAVE3 Activation through PI3K/AKT Signaling Pathway. BioMed Res. Int..

[71] Zhou Q., Yan B., Hu X., Li X.-B., Zhang J., Fang J. (2009). Luteolin inhibits invasion of prostate cancer PC3 cells through E-cadherin. Mol. Cancer Ther..

[72] Jeon Y.W., Ahn Y.E., Chung W.S., Choi H.J., Suh Y.J. (2015). Synergistic effect between celecoxib and luteolin is dependent on estrogen receptor in human breast cancer cells. Tumor Biol..

