# Supplementary material for: Synergistic Combination of Luteolin and Asiatic Acid on Cervical Cancer In Vitro and In Vivo
Source: Cancers (Basel). 2023 Jan 16;15(2):548. doi: 10.3390/cancers15020548 (PMC9857275; doi:10.3390/cancers15020548)
Supplement: Supplementary file 1 [file cancers-15-00548-s001.zip › cancers-2157470-supplementary.pdf]

Supplementary Materials:

Synergistic combination of luteolin and asiatic acid on cervical cancer cells

Ya-Hui Chen, Jyun-Xue Wu, Shun-Fa Yang, Yi-Hsuan Hsiao

Figure S1

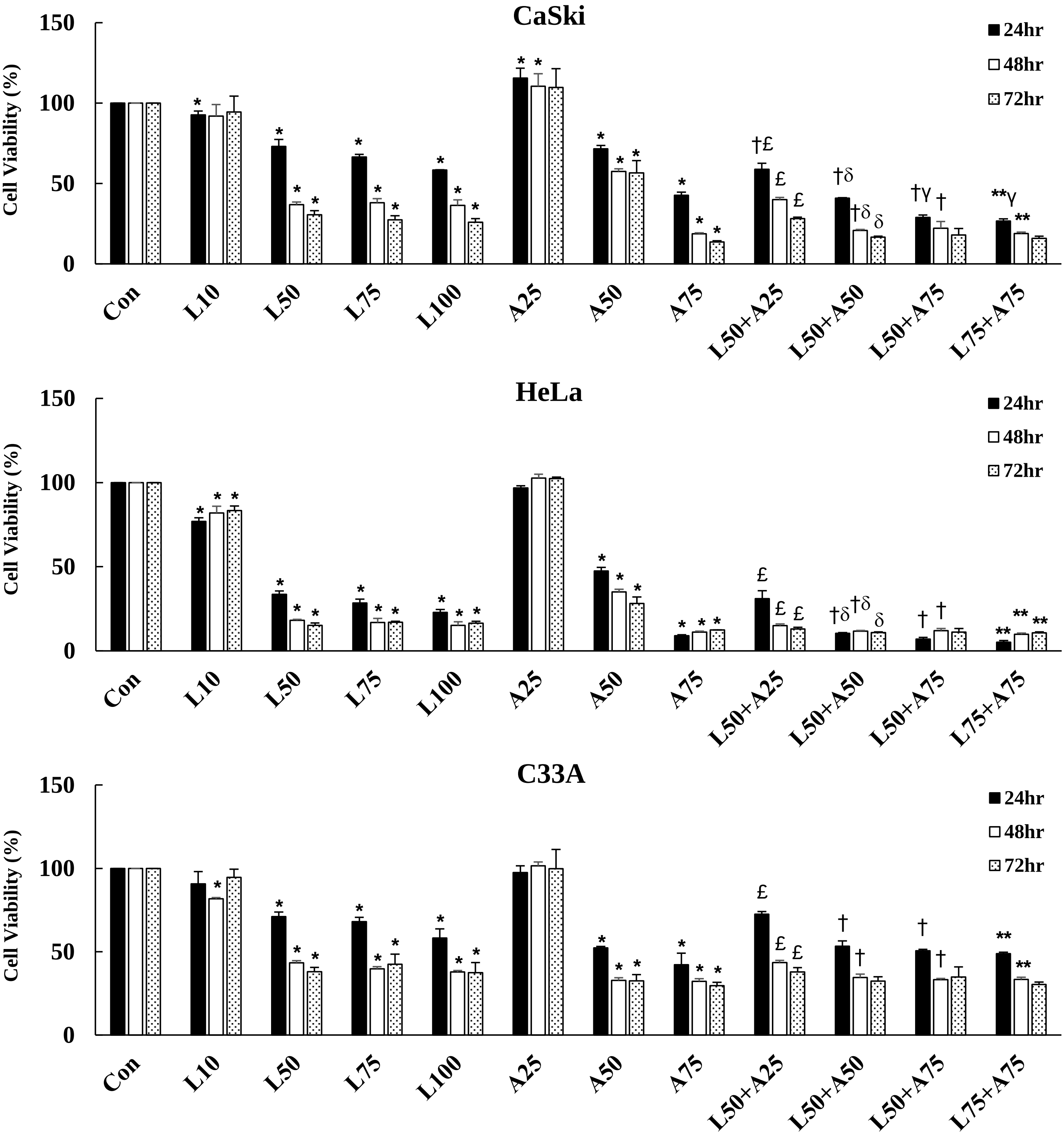
CaSki
HeLa
C33A
Cell Viability (%)
0
50
100
150
24hr
48hr
72hr
Con
L10
L50
L75
L100
A25
A50
A75
L50+A25
L50+A50
L50+A75
L75+A75

Figure S2A

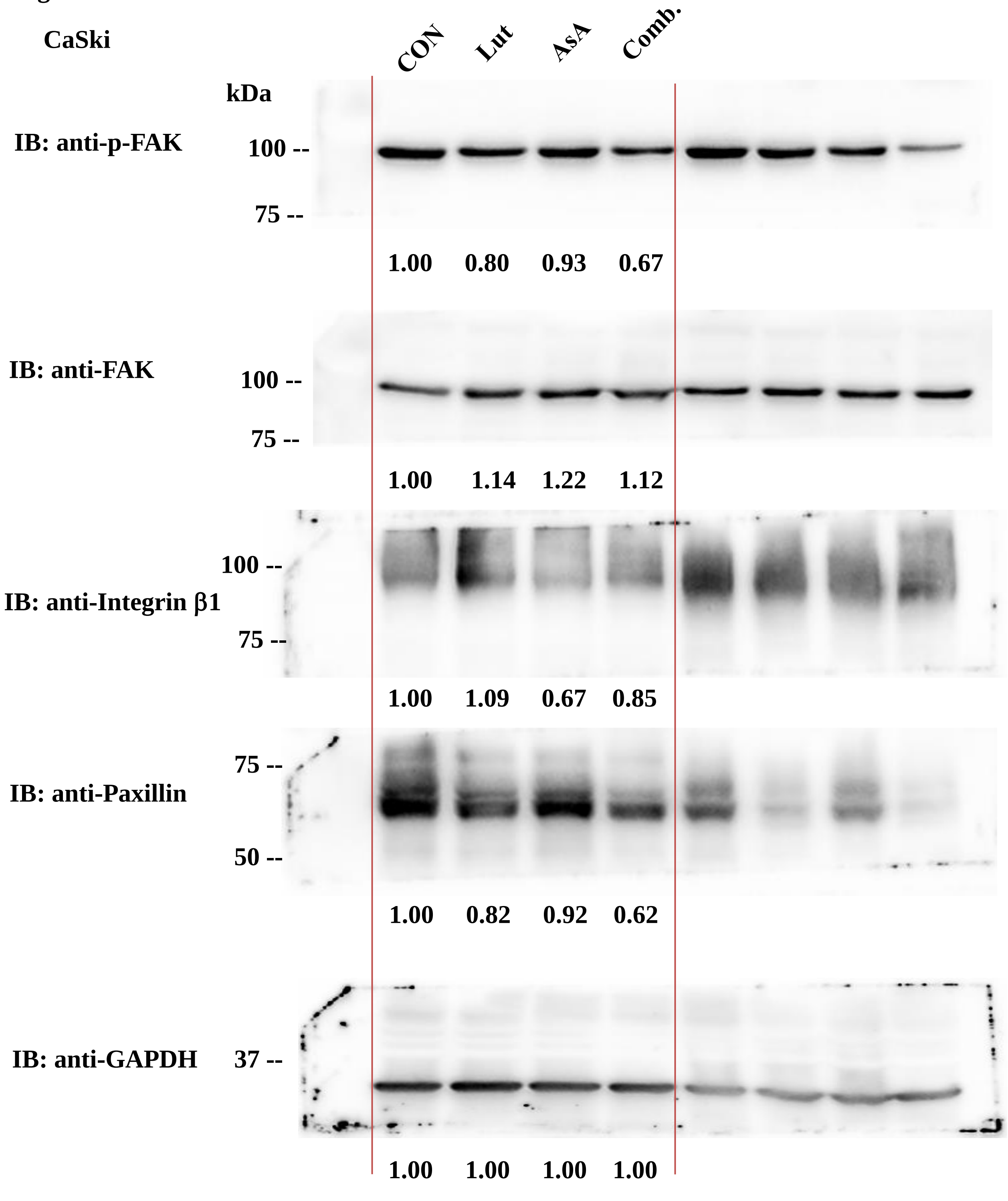
CaSki
CON
Lut
AsA
Comb.
kDa
IB: anti-p-FAK
100 --
75 --
1.00 0.80 0.93 0.67
IB: anti-FAK
100 --
75 --
1.00 1.14 1.22 1.12
IB: anti-Integrin β1
100 --
75 --
1.00 1.09 0.67 0.85
IB: anti-Paxillin
75 --
50 --
1.00 0.82 0.92 0.62
IB: anti-GAPDH
37 --
1.00 1.00 1.00 1.00

Figure S2B

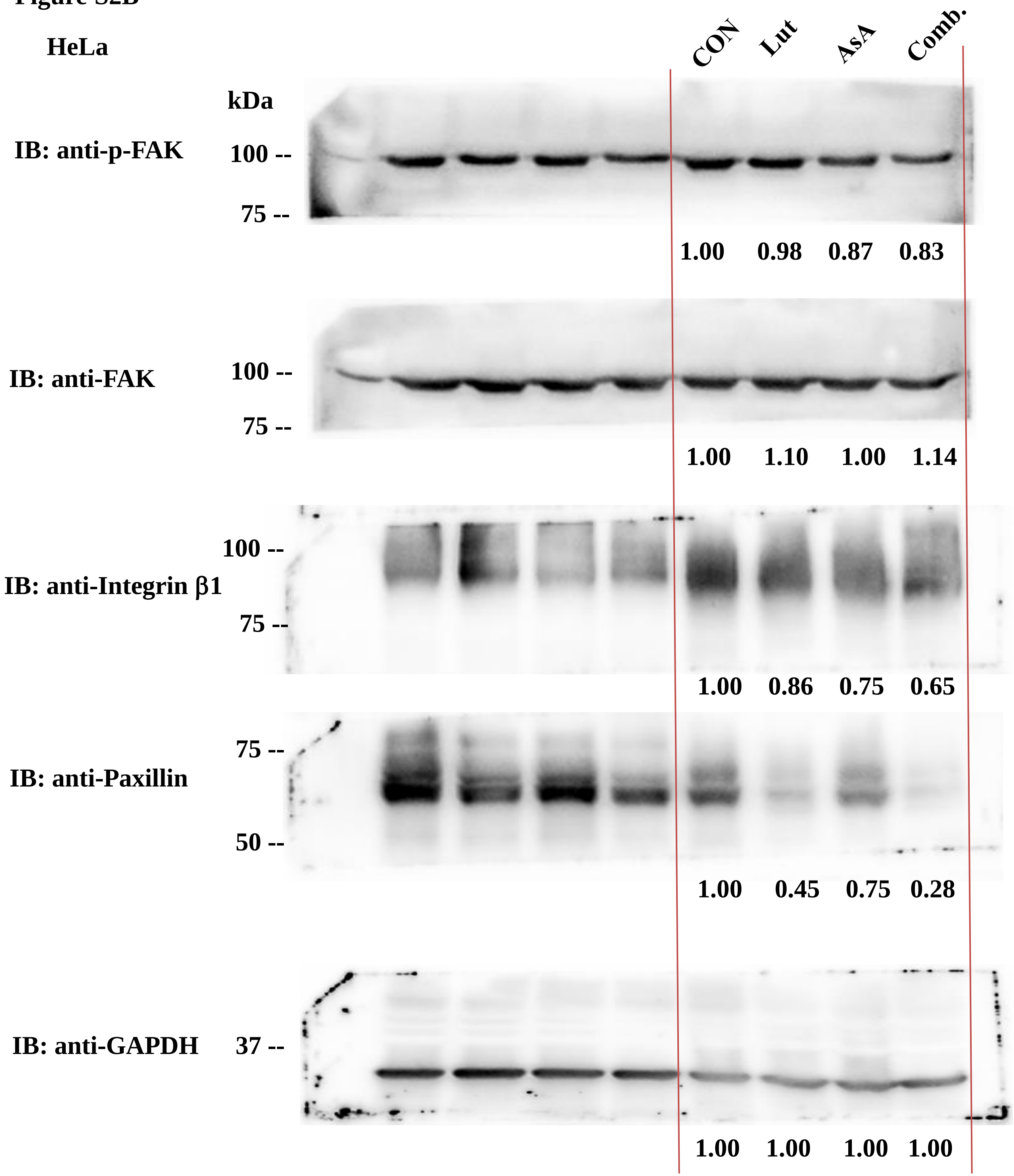
HeLa
CON
Lut
AsA
Comb.
kDa
IB: anti-p-FAK
100 --
75 --
1.00 0.98 0.87 0.83
IB: anti-FAK
100 --
75 --
1.00 1.10 1.00 1.14
IB: anti-Integrin β1
100 --
75 --
1.00 0.86 0.75 0.65
IB: anti-Paxillin
75 --
50 --
1.00 0.45 0.75 0.28
IB: anti-GAPDH
37 --
1.00 1.00 1.00 1.00

Figure S3A

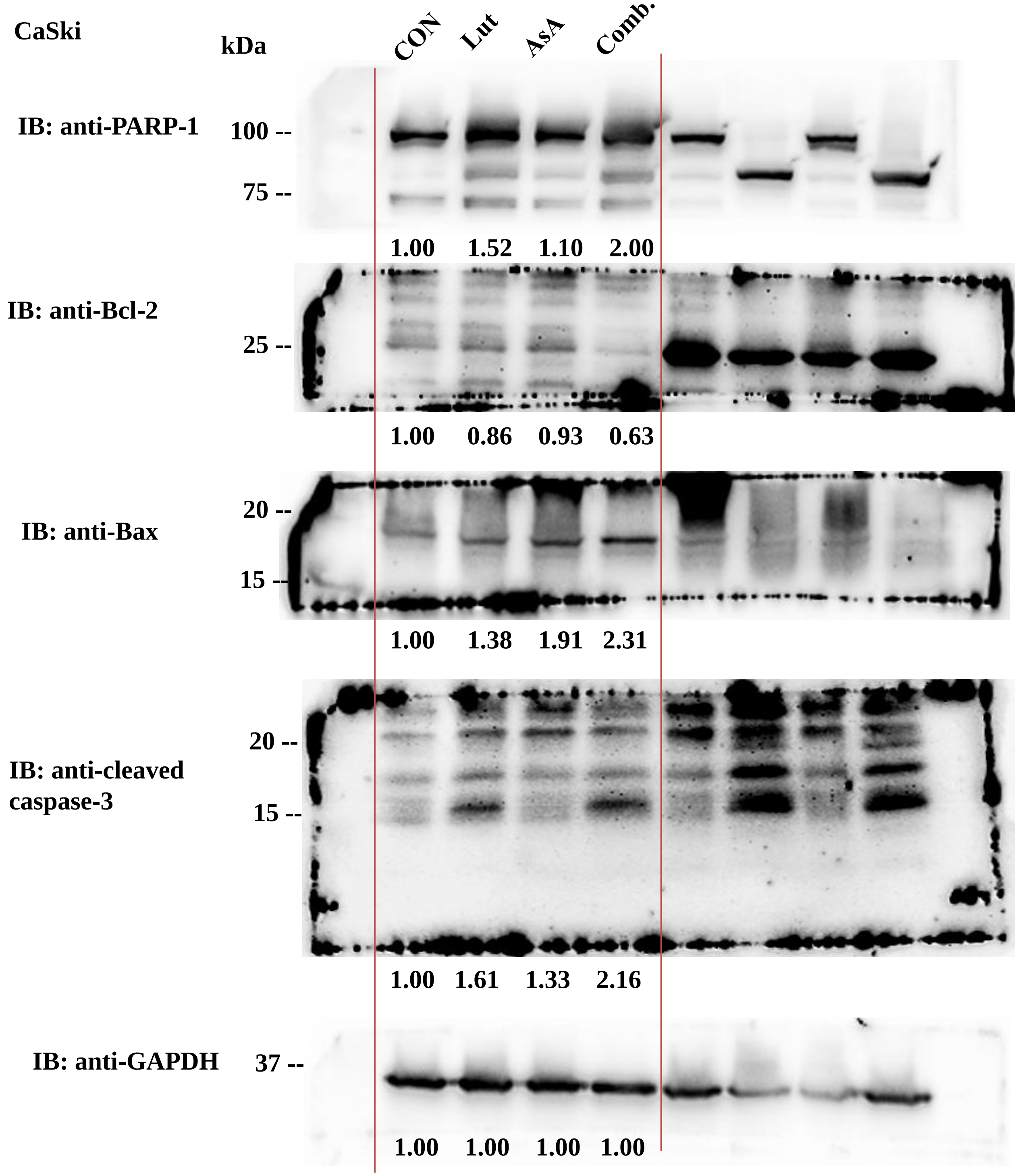
CaSki
kDa
CON
Lut
AsA
Comb.
IB: anti-PARP-1
100 --
75 --
1.00 1.52 1.10 2.00
IB: anti-Bcl-2
25 --
1.00 0.86 0.93 0.63
IB: anti-Bax
20 --
15 --
1.00 1.38 1.91 2.31
IB: anti-cleaved caspase-3
20 --
15 --
1.00 1.61 1.33 2.16
IB: anti-GAPDH
37 --
1.00 1.00 1.00 1.00

Figure S3B

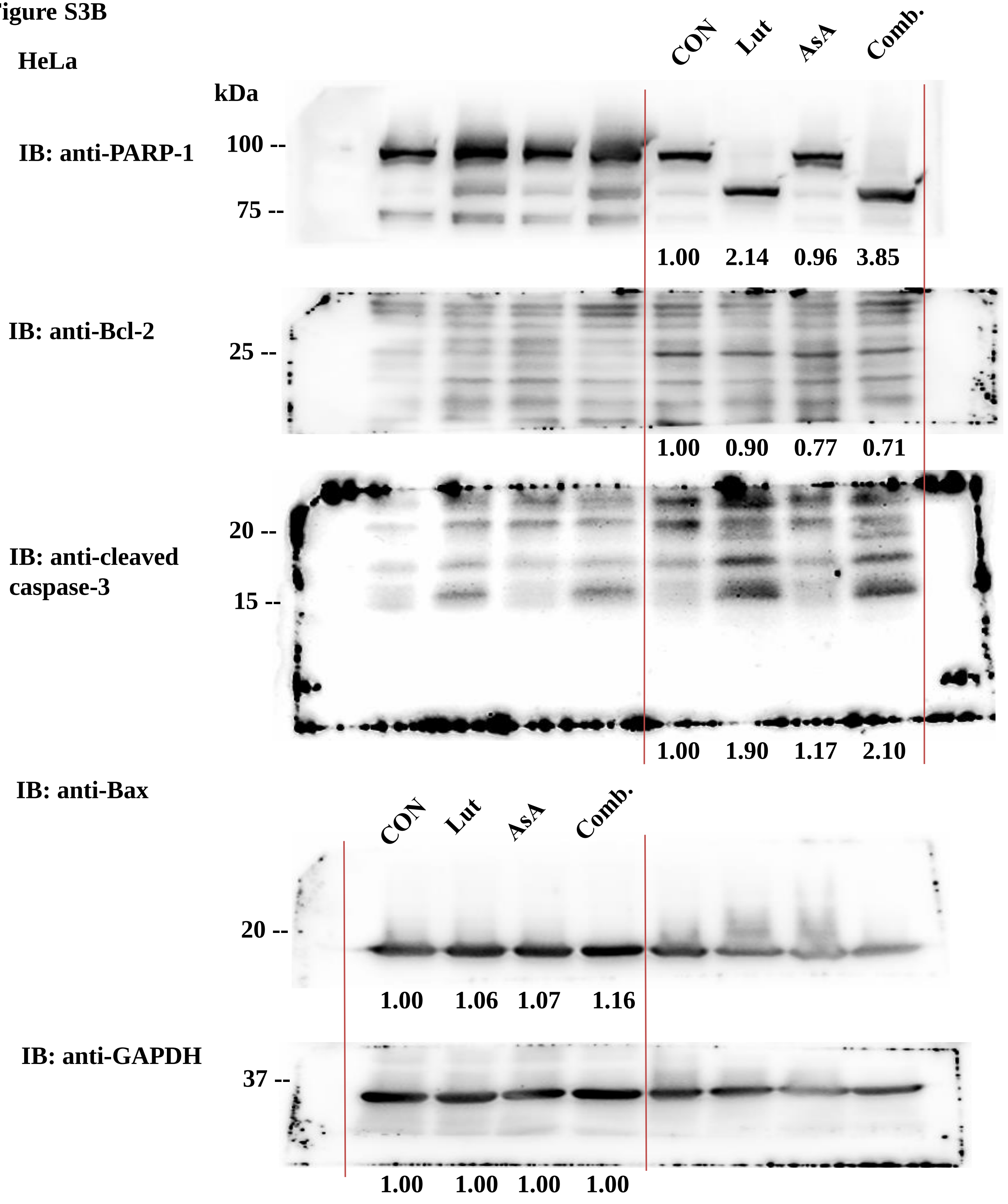
HeLa
CON
Lut
AsA
Comb.
kDa
IB: anti-PARP-1
100 --
75 --
1.00 2.14 0.96 3.85
IB: anti-Bcl-2
25 --
1.00 0.90 0.77 0.71
IB: anti-cleaved caspase-3
20 --
15 --
1.00 1.90 1.17 2.10
IB: anti-Bax
CON
Lut
AsA
Comb.
20 --
1.00 1.06 1.07 1.16
IB: anti-GAPDH
37 --
1.00 1.00 1.00 1.00

Figure S4A

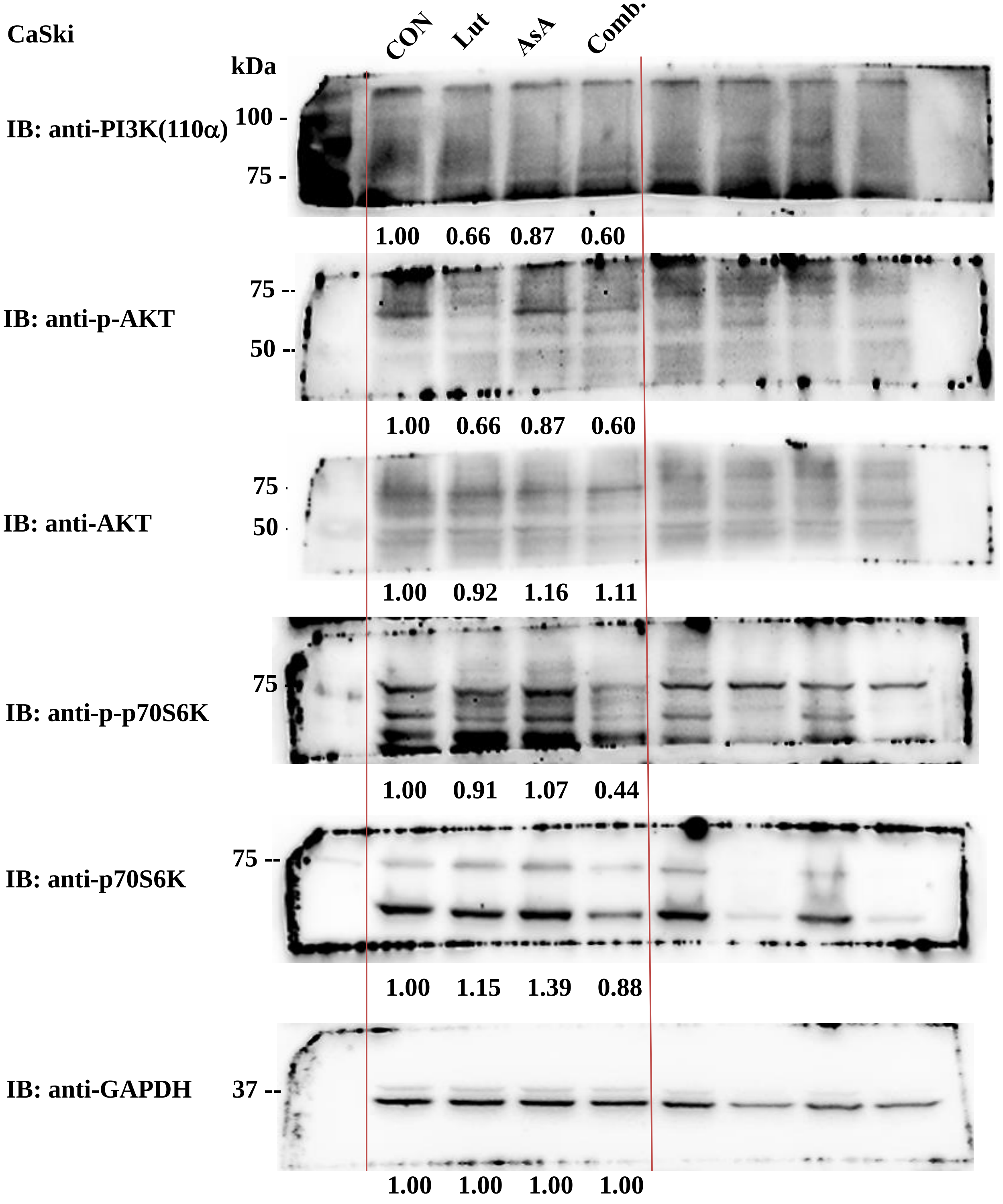
CaSki
CON
Lut
AsA
Comb.
kDa
IB: anti-PI3K(110α)
100 -
75 -
1.00 0.66 0.87 0.60
IB: anti-p-AKT
75 --
50 --
1.00 0.66 0.87 0.60
IB: anti-AKT
75
50
1.00 0.92 1.16 1.11
IB: anti-p-p70S6K
75
1.00 0.91 1.07 0.44
IB: anti-p70S6K
75 --
1.00 1.15 1.39 0.88
IB: anti-GAPDH
37 --
1.00 1.00 1.00 1.00

Figure S4B

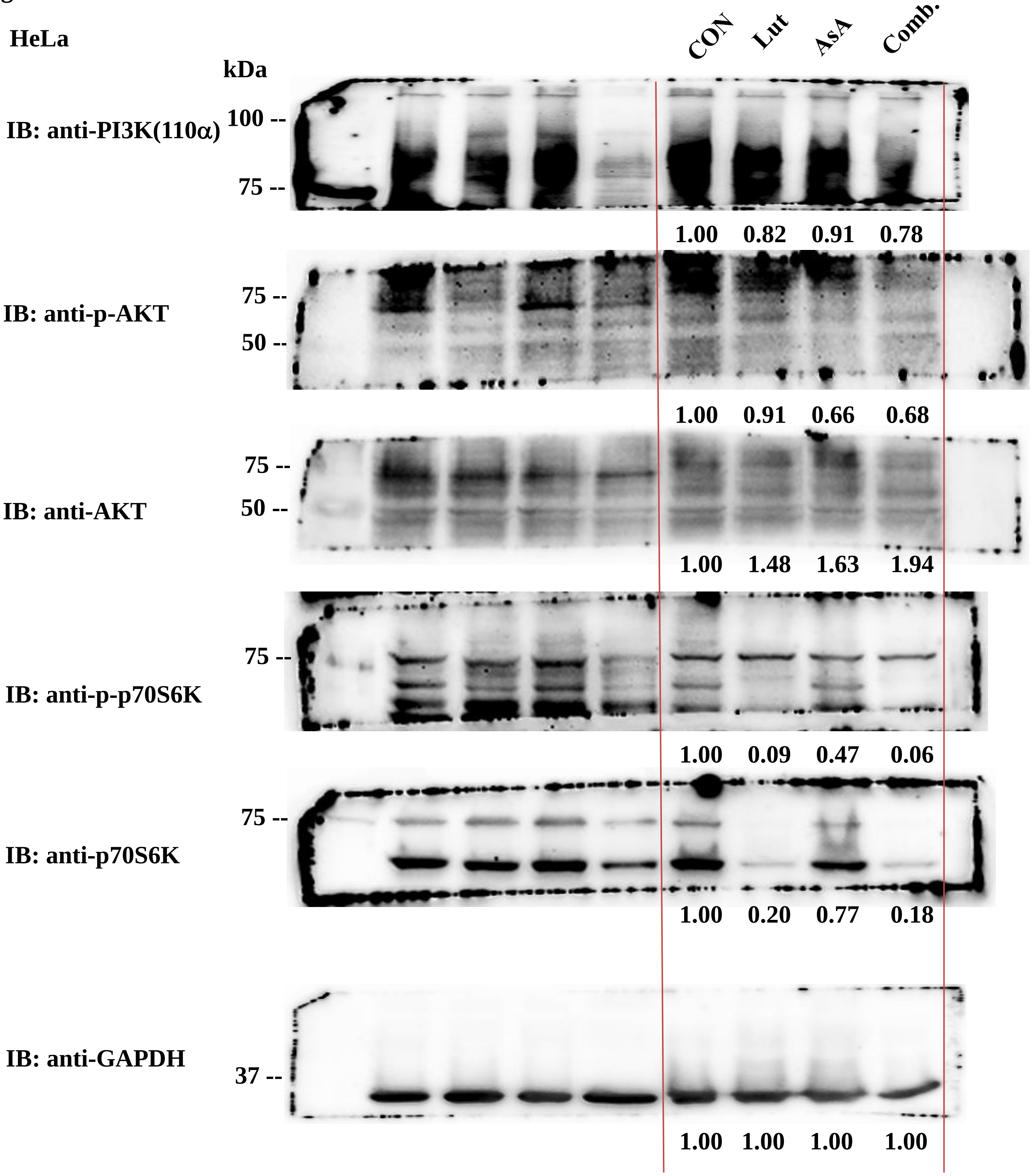
HeLa
CON
Lut
AsA
Comb.
kDa
IB: anti-PI3K(110α)
100 --
75 --
1.00 0.82 0.91 0.78
IB: anti-p-AKT
75 --
50 --
1.00 0.91 0.66 0.68
IB: anti-AKT
75 --
50 --
1.00 1.48 1.63 1.94
IB: anti-p-p70S6K
75 --
1.00 0.09 0.47 0.06
IB: anti-p70S6K
75 --
1.00 0.20 0.77 0.18
IB: anti-GAPDH
37 --
1.00 1.00 1.00 1.00

Figure S5A

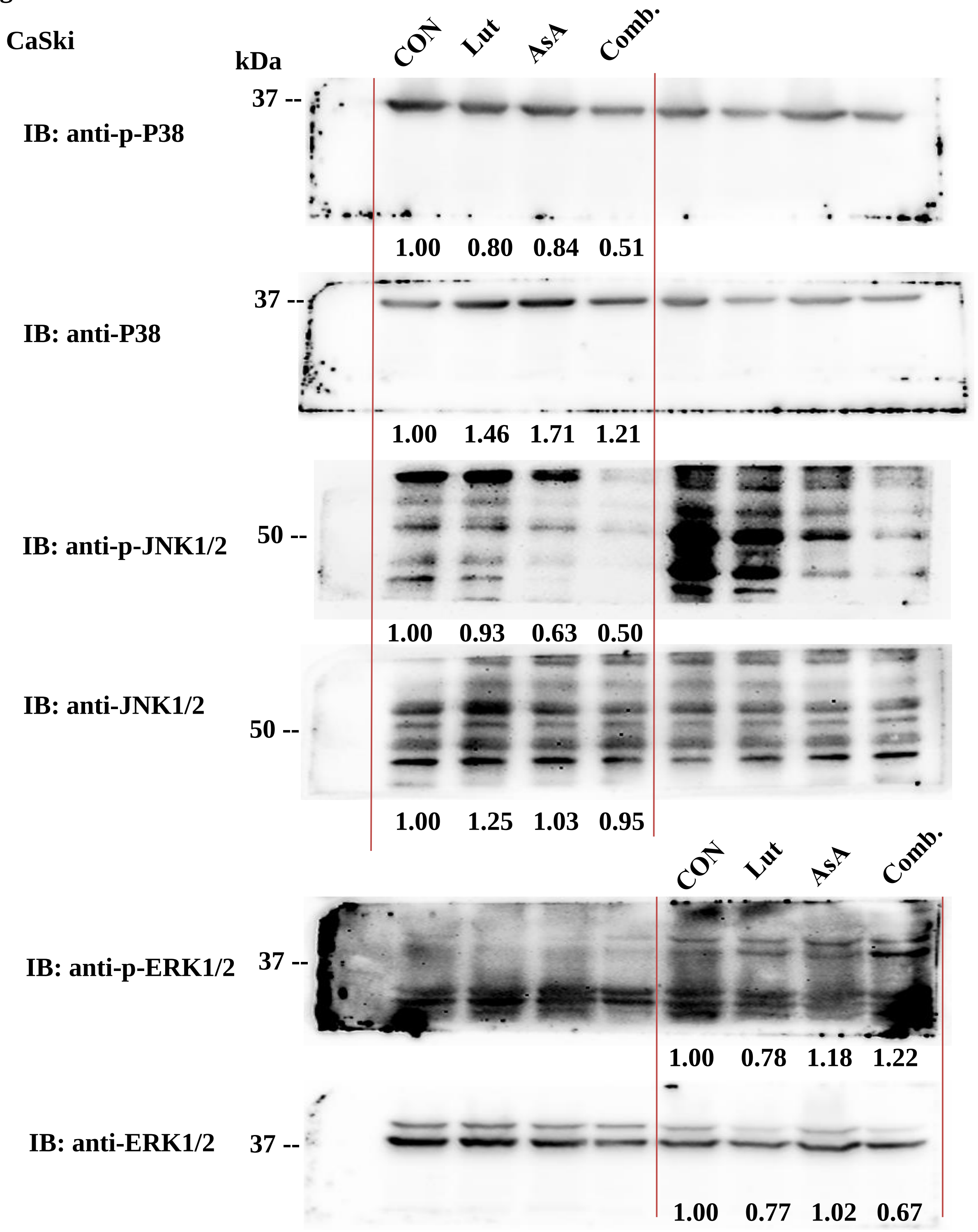
CaSki
kDa
CON
Lut
AsA
Comb.
IB: anti-p-P38
37 --
1.00 0.80 0.84 0.51
IB: anti-P38
37 --
1.00 1.46 1.71 1.21
IB: anti-p-JNK1/2
50 --
1.00 0.93 0.63 0.50
IB: anti-JNK1/2
50 --
1.00 1.25 1.03 0.95
CON
Lut
AsA
Comb.
IB: anti-p-ERK1/2
37 --
1.00 0.78 1.18 1.22
IB: anti-ERK1/2
37 --
1.00 0.77 1.02 0.67

Figure S5B

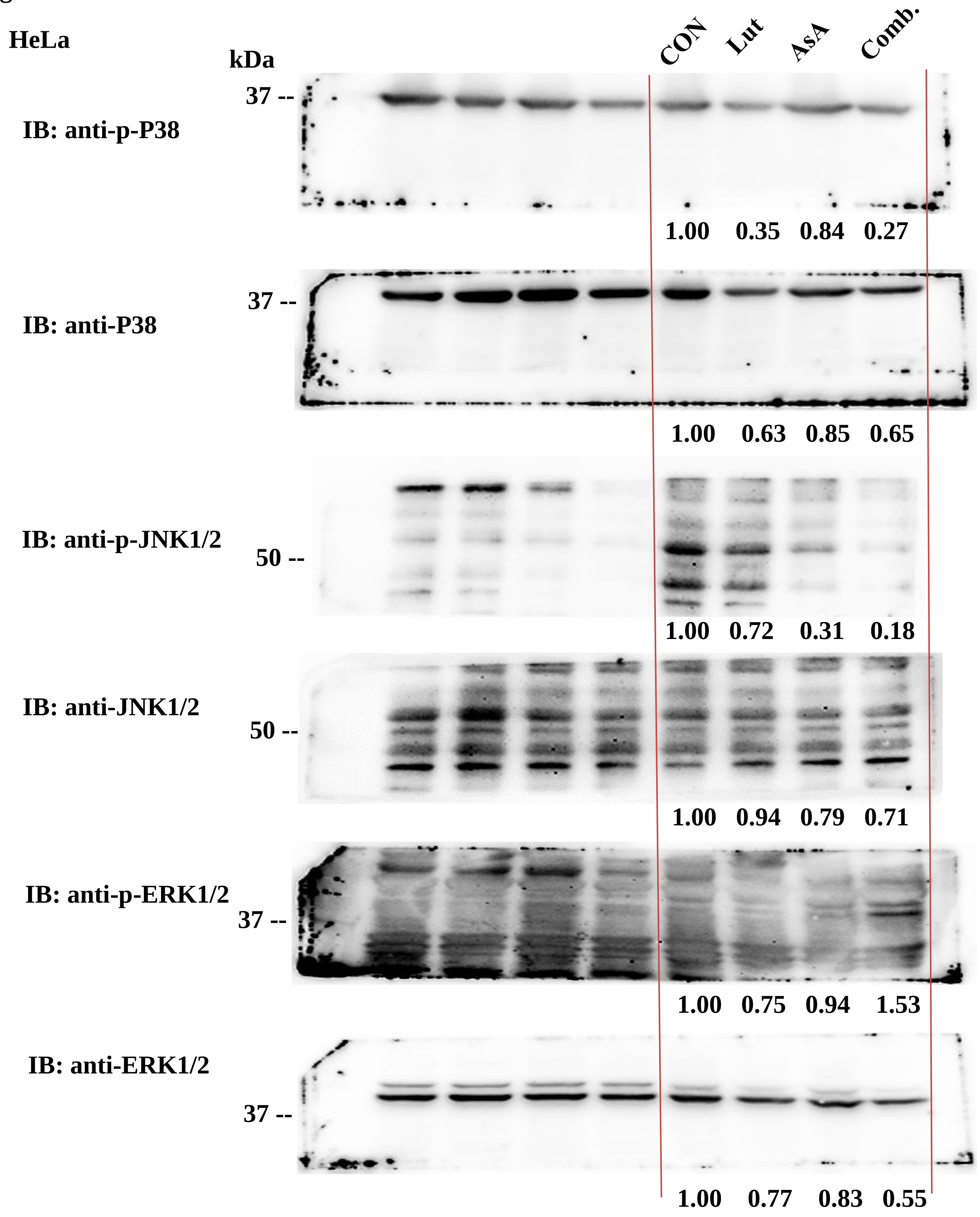
HeLa
kDa
CON
Lut
AsA
Comb.
37 --
IB: anti-p-P38
1.00 0.35 0.84 0.27
37 --
IB: anti-P38
1.00 0.63 0.85 0.65
IB: anti-p-JNK1/2
50 --
1.00 0.72 0.31 0.18
IB: anti-JNK1/2
50 --
1.00 0.94 0.79 0.71
IB: anti-p-ERK1/2
37 --
1.00 0.75 0.94 1.53
IB: anti-ERK1/2
37 --
1.00 0.77 0.83 0.55

Figure S6A

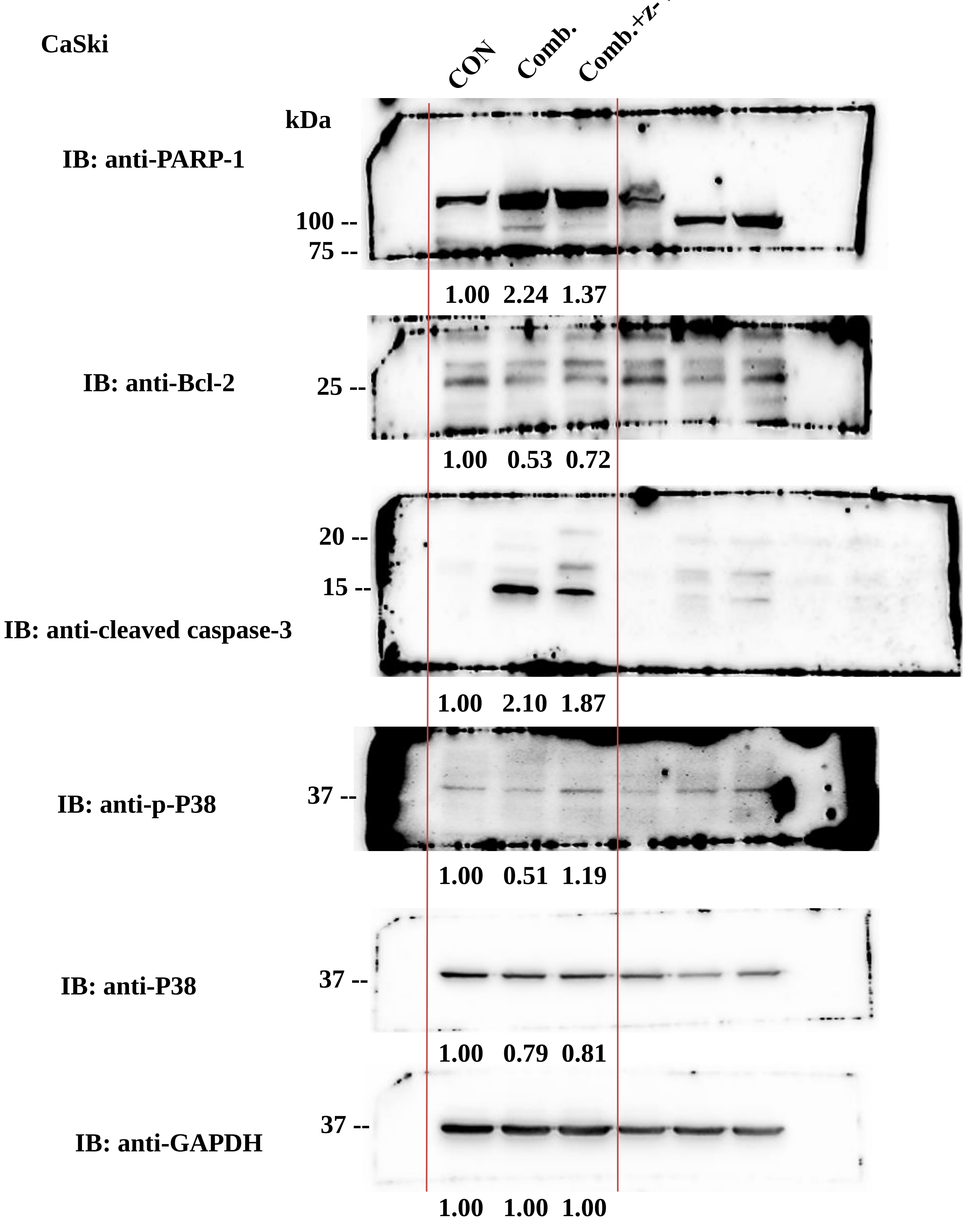
CaSki
CON
Comb.
Comb.+z-VAD
kDa
IB: anti-PARP-1
100 --
75 --
1.00 2.24 1.37
IB: anti-Bcl-2
25 --
1.00 0.53 0.72
20 --
15 --
IB: anti-cleaved caspase-3
1.00 2.10 1.87
IB: anti-p-P38
37 --
1.00 0.51 1.19
IB: anti-P38
37 --
1.00 0.79 0.81
IB: anti-GAPDH
37 --
1.00 1.00 1.00

Figure S6B

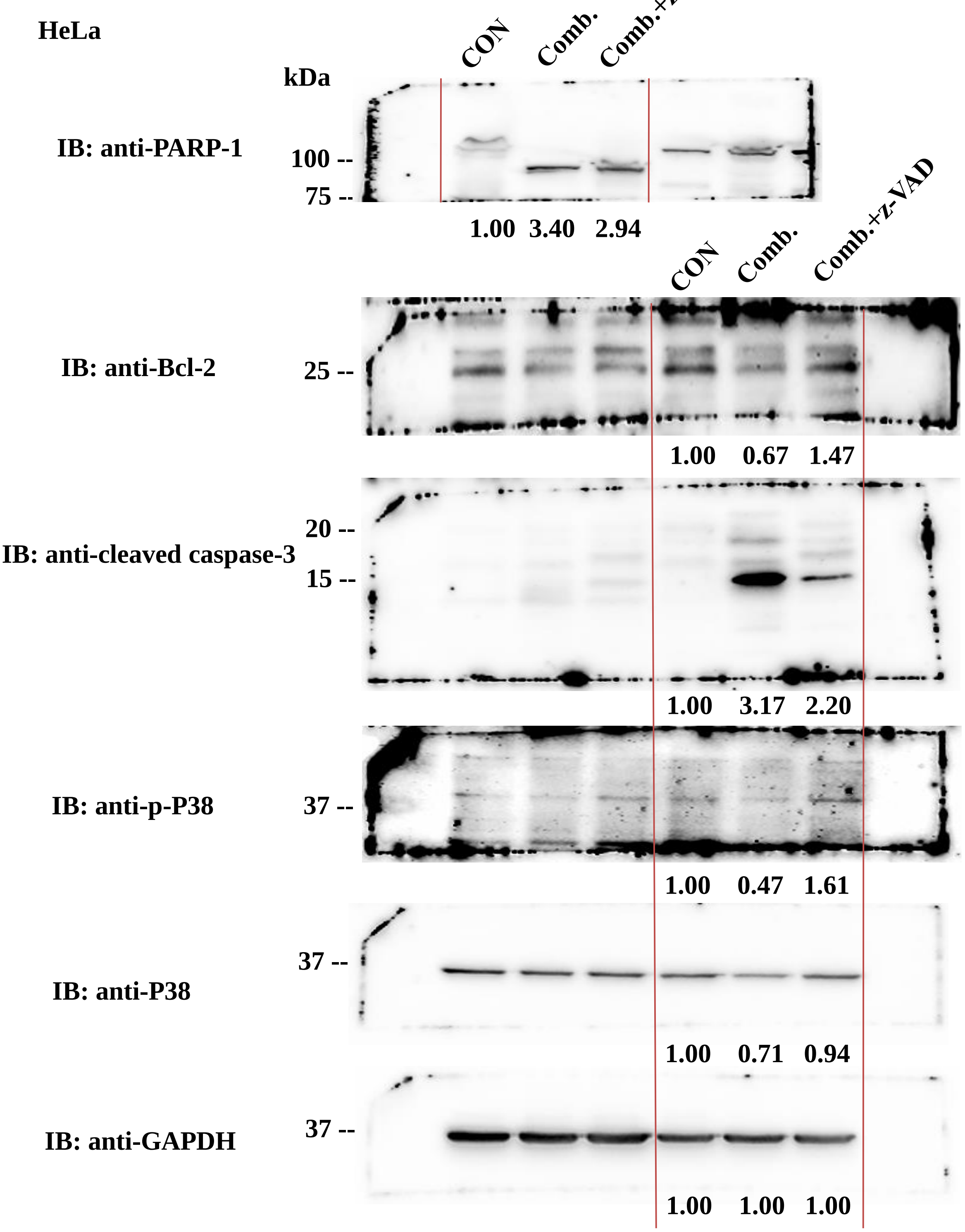
HeLa
kDa
CON
Comb.
Comb.+z-VAD
IB: anti-PARP-1
100 --
75 --
1.00 3.40 2.94
CON
Comb.
Comb.+z-VAD
IB: anti-Bcl-2
25 --
1.00 0.67 1.47
IB: anti-cleaved caspase-3
20 --
15 --
1.00 3.17 2.20
IB: anti-p-P38
37 --
1.00 0.47 1.61
IB: anti-P38
37 --
1.00 0.71 0.94
IB: anti-GAPDH
37 --
1.00 1.00 1.00
